# Metal–Support Interaction between Titanium Oxynitride and Pt Nanoparticles Enables Efficient Low-Pt-Loaded High-Performance Electrodes at Relevant Oxygen Reduction Reaction Current Densities

**DOI:** 10.1021/acscatal.3c03883

**Published:** 2024-02-02

**Authors:** Armin Hrnjić, Ana Rebeka Kamšek, Lazar Bijelić, Anja Logar, Nik Maselj, Milutin Smiljanić, Jan Trputec, Natan Vovk, Luka Pavko, Francisco Ruiz-Zepeda, Marjan Bele, Primož Jovanovič, Nejc Hodnik

**Affiliations:** † Department of Materials Chemistry, National Institute of Chemistry, Hajdrihova 19, Ljubljana 1000, Slovenia; ‡ University of Nova Gorica, Vipavska 13, Nova Gorica 5000, Slovenia; § Faculty of Chemistry and Chemical Engineering, University of Ljubljana, Večna pot 113, Ljubljana 1000, Slovenia

**Keywords:** oxygen reduction reaction, titanium oxynitride support, metal−support interaction, floating electrode, 4DSTEM

## Abstract

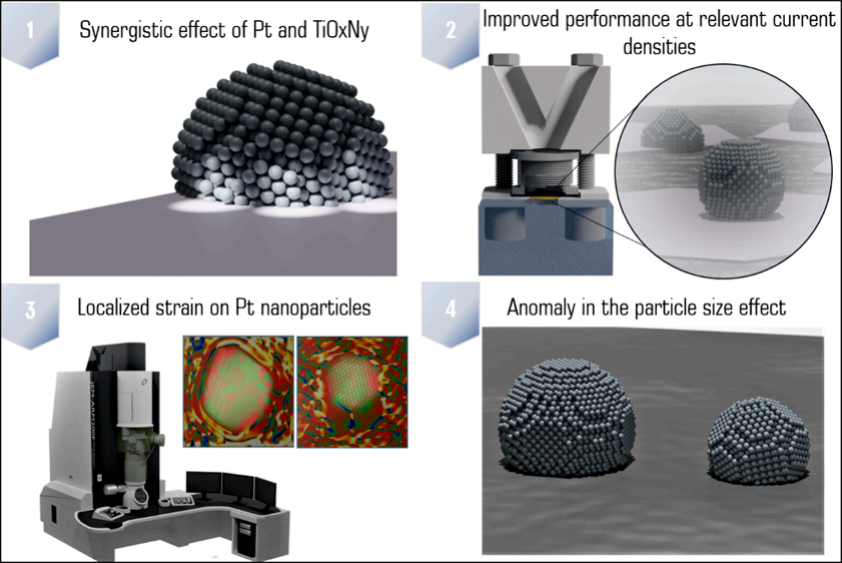

In the present work, we report on a synergistic relationship between platinum nanoparticles and a titanium oxynitride support (TiO_*x*_N_*y*_/C) in the context of oxygen reduction reaction (ORR) catalysis. As demonstrated herein, this composite configuration results in significantly improved electrocatalytic activity toward the ORR relative to platinum dispersed on carbon support (Pt/C) at high overpotentials. Specifically, the ORR performance was assessed under an elevated mass transport regime using the modified floating electrode configuration, which enabled us to pursue the reaction closer to PEMFC-relevant current densities. A comprehensive investigation attributes the ORR performance increase to a strong interaction between platinum and the TiO_*x*_N_*y*_/C support. In particular, according to the generated strain maps obtained via scanning transmission electron microscopy (STEM), the Pt-TiO_*x*_N_*y*_/C analogue exhibits a more localized strain in Pt nanoparticles in comparison to that in the Pt/C sample. The altered Pt structure could explain the measured ORR activity trend via the d-band theory, which lowers the platinum surface coverage with ORR intermediates. In terms of the Pt particle size effect, our observation presents an anomaly as the Pt-TiO_*x*_N_*y*_/C analogue, despite having almost two times smaller nanoparticles (2.9 nm) compared to the Pt/C benchmark (4.8 nm), manifests higher specific activity. This provides a promising strategy to further lower the Pt loading and increase the ECSA without sacrificing the catalytic activity under fuel cell-relevant potentials. Apart from the ORR, the platinum-TiO_*x*_N_*y*_/C interaction is of a sufficient magnitude not to follow the typical particle size effect also in the context of other reactions such as CO stripping, hydrogen oxidation reaction, and water discharge. The trend for the latter is ascribed to the lower oxophilicity of Pt-based on electrochemical surface coverage analysis. Namely, a lower surface coverage with oxygenated species is found for the Pt-TiO_*x*_N_*y*_/C analogue. Further insights were provided by performing a detailed STEM characterization via the identical location mode (IL-STEM) in particular, via 4DSTEM acquisition. This disclosed that Pt particles are partially encapsulated within a thin layer of TiO_*x*_N_*y*_ origin.

## 1 Introduction

Due to their inherently green character, i.e., converting hydrogen and oxygen while producing electricity with water and heat as the only byproducts, proton exchange membrane fuel cells (PEMFCs) have been firmly established as one of the crucial platforms in tackling the energy crisis and environmental pollution. Even though PEMFCs have made significant strides toward commercialization, further improvements, primarily concerning the cathodic oxygen reduction reaction (ORR), are needed. Within the following two segments, PEMFCs are pivotal for competition with conventional technologies. The first is the requirement for highly active and cost-effective catalysts for cathodes.^1−3^ So far, the majority of the research efforts typically evolved around carbon-supported platinum and platinum alloys.^4,5^ Primarily, the focus has been on the thorough investigation of alloying and shape control of platinum-containing catalysts with a plethora of variants markedly surpassing the required thresholds.^2,6^ Within these core-shell nanoparticles (i.e., a platinum-enriched shell and a non-noble-metal-enriched alloy core), they have received enormous attention. Here, experimental and theoretical investigations suggested that core-shell structures are beneficial for ORR kinetics because of the modified d-band center of surface Pt atoms caused by the underlying alloying effect.^7−10^ However, the ORR activity descriptors need to be synchronized by stability counterparts in order to come up with activity–stability relations, i.e., prerequisite criteria for PEMFC implementation. Indeed, in recent years, the efforts to pursue such relations have intensified^11^ identifying platinum dissolution and carbon support corrosion as the two major primary degradation mechanisms of the ORR catalyst layer.^12,13^ Carbon corrosion may induce secondary degradation mechanisms of platinum particle detachment or agglomeration and additionally cause increased mass transport resistance of reactant gases and water transport issues.^14−16^ This may be completely avoided through the use of alternative supports, such as corrosion-resistant transition metal oxides (e.g., TiO_2_, SnO_2_, SiO_2_, or WO_*x*_).^17−19^ Their use as supporting materials for noble-metal electrocatalysts in PEMFCs has not received much attention, especially at the industry level, because of their typically low electronic conductivity and challenges associated with synthesizing a sufficiently high surface area. Nevertheless, these classes of supports are highly appealing^20,21^ due to their chemical stability and durability under the operating conditions of PEMFCs, i.e., oxidative and strongly acidic conditions. Apart from contributing to the durability of the catalyst layer, the support can increase the intrinsic catalytic activity by a so-called strong metal–support interaction (SMSI), via electronic or geometric effects. The SMSI can potentially manifest itself in different ways to improve ORR activity such as modification of the electronic states or the Fermi level of Pt that pushes the formation of Pt–OH groups to higher potentials,^22,23^ spillover of OH_ad_ groups onto the support, and reduction of OH coverage by lateral repulsion between Pt–OH and oxide surfaces.^24−26^ Additionally, as shown recently, oxygen defects from the oxide support can induce direct interaction with platinum.^27,28^ More specifically, electron transfer from oxygen defects or the support cation to Pt resulted in the continual metallic state of Pt, leading to the decreased adsorption of oxygenated species on the Pt surface. Note that the Pt–OH bond strength is a crucial activity descriptor for ORR,^29−32^ emphasizing that the interaction between electrocatalytically active Pt particles and the oxide support could ultimately enable the design of more efficient catalysts.

However, one should keep in mind that the potential influence of Pt–support interactions on ORR was almost exclusively studied via the rotating disk electrode (RDE) technique where due to low oxygen mass transport, only potentials above 0.8 V vs RHE are accessible for kinetic analysis.^33^ This is preventing one from obtaining comprehensive ORR trends, and instead, performance is then typically extrapolated to industry-relevant lower potentials. This adds great uncertainty to the validity of predictions as the postulate that ORR current follows a single exponential increase (i.e., a single Tafel slope) with overpotential in the Pt oxide-free region might not hold.^34−36^ Indeed, as shown in recent studies based on kinetic modeling and floating electrode technique (FET) measurements,^36,37^ the site blockage of reaction intermediates, and not the mass transport resistance as typically assumed,^38−42^ plays a decisive role at potentials below 0.8 V. This clearly necessitates the employment of electrochemical setups enabling the analysis at high polarization, i.e., high current density measurements to observe platinum–support interactions credibly. Accordingly, within this paper, we employ a modified floating electrode (MFE) to assess the ORR performance of carbon (Pt/C) and titanium oxynitride-supported Pt nanoparticles (Pt-TiO_*x*_N_*y*_/C) across a wide, PEMFC-relevant potential window. The obtained electrocatalytic trends reveal anomalous behavior of the platinum surface if in configuration with the TiO_*x*_N_*y*_/C support. Namely, despite the smaller particle size and larger electrochemical surface area (ECSA), the intrinsic ORR performance at low potentials is substantially higher in comparison to larger particles (Pt/C), which should in principle be useful to develop more efficient low-Pt-loaded high-performance electrodes.^38^

## 2 Experimental Section

### 2.1 Synthesis

We emphasize that the synthesis of the Pt-TiO_*x*_N_*y*_/C composite investigated herein is described in detail in our recent publication (see also SI Section S1).^43^ The final Pt-TiO_*x*_N_*y*_/C material contained 18.4 wt % Pt, according to the ICP-OES analysis.^43^ A commercial Pt/C analogue (TEC10E50E-HT, TKK, Japan) was selected for comparison and contained 50.6 wt % Pt.

### 2.2 Structural Characterization

A Pt-TiO_*x*_N_*y*_/C composite was characterized by XRD and TEM. The XRD pattern was recorded using a D4 Endeavor, Bruker AXS diffractometer with Cu Kα radiation (λ = 1.5406 Å) and a Sol-X energy-dispersive detector. A step of 0.034° and a holding time of 100 s were used during XRD acquisition. For the detailed microstructural investigation, a Cs probe-corrected scanning transmission electron microscope (Jeol ARM 200 CF) with an attached Jeol Centurio EDXS system with a 100 mm^2^ SDD detector and a Gatan Quantum ER DualEELS system was used. For comparison, the same structural characterization was also performed for a commercial Pt/C catalyst (TEC10E50E-HT, TKK, Japan).

#### 2.2.1 Identical Location Scanning Transmission Electron Microscopy (IL-STEM)

IL-STEM characterization was performed before and after electrochemical perturbation (EP) of the Pt-TiO_*x*_N_*y*_/C-coated TEM grid under the MFE configuration. EP consisted of potentiodynamic cycling between 0.05 and 1.2 V_RHE_ (200 cycles, 300 mV s^–1^) under the argon atmosphere. Different regions were imaged using the annular dark-field (ADF) and bright-field (BF) detectors, but in addition, some of the areas were also imaged using a pixelated detector MerlinEM for 4DSTEM acquisition. Virtual detectors were then used to generate BF images by using a mask around the central diffraction disk and an annular mask excluding just the central diffraction disk for ADF images. This allowed us to enhance the contrast of some features in the images by selecting a larger range in the reciprocal space.

#### 2.2.2 Strain Analysis

Atomically resolved high-angle annular dark-field (HAADF) STEM images of Pt nanoparticles, collected as stacks of 10 frames, were used for a two-part strain analysis. Frames were aligned by using a rigid registration algorithm to improve the signal-to-noise ratio and eliminate any possible drift effects. Pt atomic column positions were determined using an in-house algorithm, which consisted of a preprocessing step to enhance the signal coming from the nanoparticle with respect to the support and background, then calculating centers of mass of individual columns based on intensities, and lastly fitting 2D asymmetric Gaussian distributions onto individual columns using centers of mass as an initial guess. The obtained column positions were used as seeds for constructing a Voronoi diagram. Voronoi cells were then colored according to their area, relative to the average cell area of each respective nanoparticle, to highlight any systematic anomalies in the unit cell size. Independently of that analysis, geometric phase analysis (GPA) was conducted to confirm the strain effects, generating strain maps using the information in the frequential domain rather than relying on using atomic column positions in real space.^44^

### 2.3 Electrochemical Measurements

#### 2.3.1 TF-RDE Measurements

Experiments were carried out using a thin-film rotating disk electrode (TF-RDE) in a standard three-compartment cell. The reference electrode used was a reversible hydrogen electrode (Gaskatel HydroFlex), and a graphite rod served as the counter electrode. Before being applied to the glassy carbon (GC) electrodes, the catalyst suspensions were sonicated for 20 min. In the case of the Pt-TiO_*x*_N_*y*_/C sample, the suspension was prepared by mixing 5.23 mg of the catalyst powder, 1.960 mL of isopropyl alcohol, 0.650 mL of ultrapure Milli-Q water (18.2 MΩ cm), and 52 μL of Nafion solution (5 wt %) resulting in a catalyst concentration of 2 mg mL^–1^. In the case of a commercial Pt/C analogue (TEC10E50E-HT, TKK, Japan), the suspension was prepared by mixing 5 mg of the catalyst powder, 2.25 mL of isopropyl alcohol, 0.75 mL of ultrapure Milli-Q water (18 MΩ cm), and 54 μL of Nafion solution (5 wt %) resulting in a catalyst concentration of 1.66 mg mL^–1^. The measurements were performed using a Biologic SP-300 potentiostat. Measurements were performed in 0.1 mol L^–1^ HClO_4_ (ROTIPURAN Supra, 70%). Before measurements, the electrode was activated using cyclic voltammetry (CV) with 200 cycles at a scan rate of 300 mV/s and a rotation rate of 600 rpm, ranging from 0.05 V vs RHE to 1.2 V vs RHE in an Ar atmosphere. CV in the argon atmosphere was then performed at a scan rate of 20 mV/s, ranging from 0.05 V vs RHE to 1.0 V vs RHE, with a rotation rate of 1600 rpm. ORR polarization curves were obtained by performing linear scanning voltammetry under the same conditions in an oxygen atmosphere. CO stripping was performed by holding the potential of the working electrode at 0.05 V vs RHE and purging CO gas for 20 s followed by purging Ar gas for 10 min to remove any excess CO from the electrolyte. CV was then performed to oxidize (strip) the adsorbed CO. Before the CO stripping experiment (only for the case of the Pt-TiO_*x*_N_*y*_/C sample), the so-called CO stripping simulation (COSS) experiment was performed following the procedure from ref (45). Due to the redox properties of TiO_*x*_N_y_, this measure turned out to be essential to correctly isolate the CO stripping current response from the background and hence accurately determine the Pt electrochemically active surface area (see SI Section S5). Both for the TF-RDE and the MFE experiments, the working electrode was set to 0.05 V vs RHE for 20 s in the Ar atmosphere instead of the CO atmosphere. For TF-RDE, this was followed by an additional 10 min of argon purging, and for the MFE, argon gas was purged for 2 min in the same manner as in the CO stripping experiment. Afterward, CV was performed to simulate electrochemical conditions under CO stripping. This CV was used as a background to be subtracted from the CO stripping signal (performed after the COSS). The ORR polarization curves were measured under *iR* compensation via positive feedback (85% was compensated for). The hydrogen oxidation reaction (HOR) was used to probe the possible formation of a thin film of a TiO_*x*_N_*y*_ overlayer on the surface of Pt nanoparticles, which may be formed either during synthesis or during electrochemical degradation. For this purpose, GC electrodes were covered with 5 μL of a Pt-TiO_*x*_N_*y*_/C catalyst suspension, and the same activation procedure was applied. To check the possible formation of the TiO_*x*_N_*y*_ overlayer during extensive electrochemical biasing, the Pt-TiO_*x*_N_*y*_/C sample was subjected to extensive cycling (5000 CV scans, sweep rate of 300 mV/s, Ar-saturated 0.1 M HClO_4_) in the potential range of 0.60–0.95 V vs RHE. HOR polarization curves were collected both before and after the degradation test in the hydrogen-saturated 0.1 M HClO_4_ electrolyte (a scan rate of 10 mV/s, a potential window between 0.05 V vs RHE and 1.25 V vs RHE, and a rotation rate of 1600 rpm). The HOR polarization curves were measured under *iR* compensation via positive feedback (85% was compensated for).

#### 2.3.2 Adsorbate Coverage Determination

Voltammetric experiments for the apparent surface coverage determination were performed in a two-compartment glass cell separating the reference electrode (Ag/AgCl in 3 M NaCl) from the working electrode (glassy carbon disk substrate, GC, *d* = 2 mm, Metrohm) via the Luggin capillary and the electrolyte bridge. Potentials were later converted to reference the RHE electrode. A graphite rod was used as the counter electrode. The GC electrode was polished on a 0.5 μm polishing slurry on a TriDent polishing cloth (Buehler, USA) prior to the application of the catalyst suspension. Before being applied to the GC electrodes, the suspensions were sonicated for 20 min. The same ink compositions as described above (see section TF-RDE Measurements) were used. Two μL of the catalyst suspension was drop cast on the GC electrode in both cases. Measurements were performed in 0.1 mol L^–1^ HClO_4_ (ROTIPURAN Supra, 70%). Before apparent surface coverage measurements, the working electrode was cycled from 0.05 to 1.2 V vs RHE with a scan rate of 300 mV s^–1^ for 100 cycles under an Ar-saturated atmosphere in order to obtain a stable cyclic voltammogram, namely, a reproducible starting state of the electrocatalyst. The system was then subjected to cyclic voltammetry measurements at varied scan rates (10, 20, 50, 100, and 200 mV s^–1^ under an Ar-saturated atmosphere; 5 cycles were performed for each respective scan rate) from which the apparent surface coverage was determined as follows. Apparent surface coverage for the two analogues was determined based on differential coefficients of electrochemical adsorption isotherms, defined as *I*(*E*)/*v* (*v* is the sweep rate, i.e., d*E*/d*t*), as a function of applied potential.^46^ The integral form, i.e., integrated charge curves (eq 1), obtained under different scan rates was used herein.^47^ Prior to integration, the curves were corrected for the background current from the double-layer capacitance. The apparent surface coverage (θ) was evaluated using eq 1 and by calculating *Q*/*Q*_H_. The *Q*_H_ parameter corresponds to the charge associated with hydrogen underpotential deposition and desorption (HUPD) and corresponds to the total Pt surface coverage. The *Q*_H_ was determined from the 100 mV s^–1^ measurement.
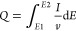
1

#### 2.3.3 Modified Floating Electrode Measurements

**Figure 1:**
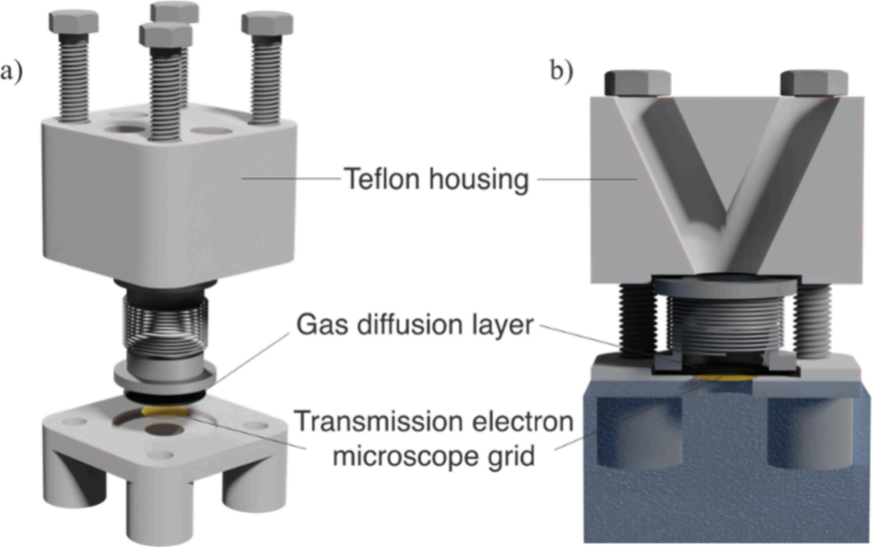
(a) Constituent parts of the MFE. (b) MFE positioning in the electrochemical cell.

A modified floating electrode setup introduced by our group was employed for ORR performance characterization under elevated mass transport of O_2_ (Figure 1).^48,49^ All MFE experiments were performed in the one-compartment Teflon cell with a 1 mol L^–1^ HClO_4_ (ROTIPURAN Supra, 70%) electrolyte with the conventional three-electrode system controlled by a potentiostat (Biologic SP-300). A reversible hydrogen electrode (Gaskatel HydroFlex) and Pt foil were used as a reference and counter electrode, respectively. The catalyst ink was sonicated and then placed on the gold TEM grids (PELCO, center-marked, 300 mesh) that serve as a working electrode. Initially, the so-called “break-in” conditioning was employed for the formation of proton transport pathways to the catalyst layer.^50^ The “break-in” (depicted in SI Section S3) consisted of the potential cycling in hydrogen and oxygen atmospheres, i.e., performing hydrogen oxidation and hydrogen evolution reaction (HOR/HER) scans followed by the ORR scan. To avoid mixing the two gases, argon was purged in between the scans. Cyclic voltammetry in the argon atmosphere and CO stripping were performed for the estimation of the Pt electrochemical surface area of catalysts. CO stripping was performed by holding the potential of the working electrode in a similar manner to that in the TF-RDE (see section TF-RDE Measurements), with the only difference being the 2 min purging of the Ar gas instead of 10 min, which is normally used for the TF-RDE measurements. The time was shorter because the MFE is much more efficient in exchanging gases in the electrolyte than the RDE. Afterward, the ORR polarization curves were measured using linear sweep voltammetry (LSV) with a scan rate of 20 mV/s in the oxygen atmosphere. The ORR polarization curves were measured under *iR* compensation via positive feedback (85% was compensated for). For each of the two samples, the investigated ORR performance as a function of the catalyst loading was measured prior to direct comparison. This was to determine the limiting cases for MFE measurements (see SI Section S6). Identification of this particular regime (i.e., loading-independent ORR_spec_ regime) is necessary for the intrinsic comparison of different ORR catalysts (i.e., without additional mass transport effects).^37^ The electrolyte resistance was determined from electrochemical impedance spectroscopy (EIS) measurement performed between 100 kHz and 1 Hz with an amplitude of 10 mV, where the resistance was measured using the high-frequency intercept of an impedance scan. The same ink compositions as described above (see section TF-RDE Measurements) were used.

## 3 Results and Discussion

### 3.1 Structural Description of Pt-TiO_*x*_N_*y*_/C

**Figure 2:**
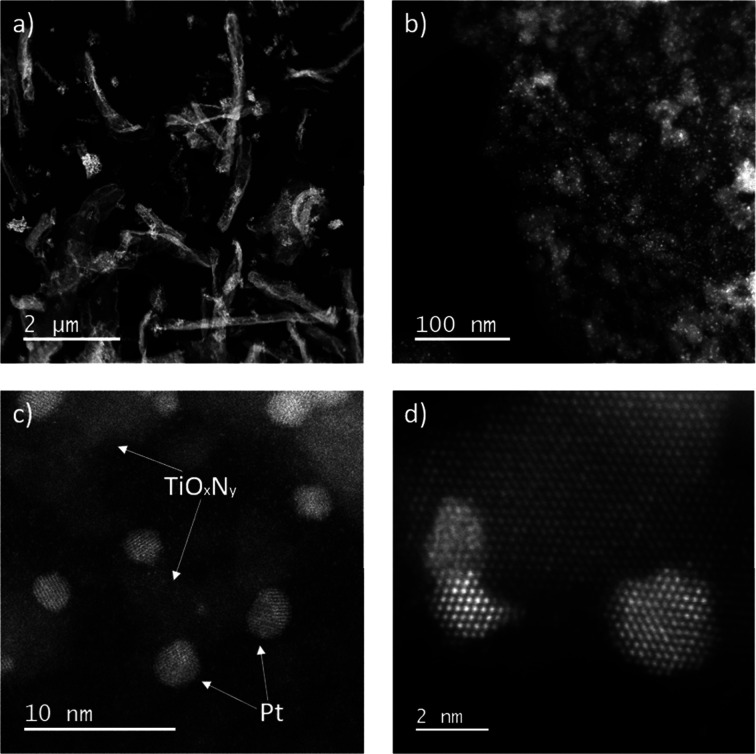
STEM-HAADF images of the Pt-TiO_*x*_N_*y*_/C sample at low and high magnifications. (a) The nanotubular structure of the carbon-supported TiO_*x*_N_*y*_ nanoribbons can be appreciated by the peculiar morphology. (b) Side-edge of a carbon nanoribbon where TiO_*x*_N_*y*_ island-like structures can be distinguished on carbon due to the *Z*-contrast. The small bright spots correspond to Pt nanoparticles. (c) Pt nanoparticles sitting on TiO_*x*_N_*y*_ island-like structures. (d) Zone-axis-oriented Pt nanoparticles sitting at the edge of a TiO_*x*_N_*y*_ island-like structure.

**Figure 3:**
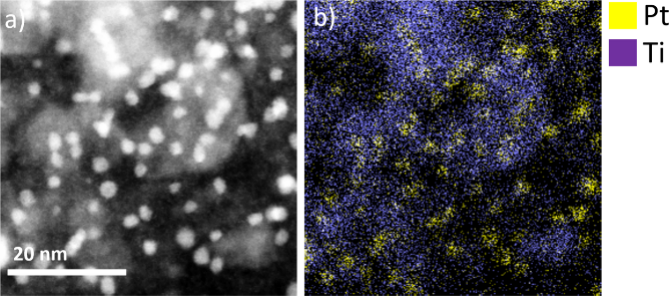
(a) STEM-HAADF image and (b) EDXS map of the Pt-TiO_*x*_N_*y*_/C sample corresponding to Pt M and Ti K signal.

The Pt-TiO_*x*_N_*y*_/C sample is composed of a combination of carbon phases obtained from graphene oxide nanoribbons (GONR), titanium oxide (TiO_*x*_N_*y*_), and platinum nanoparticles. The carbon phase has an elongated structure similar to that of carbon nanotubes with an aspect ratio of a few μm in length and a few 100 nm in width (Figure 2a). It functions as both an efficient electronic conductor (wiring) and a template for dispersing a high-surface-area ceramic support. TiO_*x*_N_*y*_ covers the surface of the GONRs in an island-like way and ranges from 5 to 20 nm in size (Figure 2b and Figure S2), with thicknesses varying from 3 to 10 nm depending on their size (Figure S3). These TiO_*x*_N_*y*_ nanostructures provide a highly dispersed support for Pt nanoparticles anchoring, which are attached to the TiO_*x*_N_*y*_ or its edges (Figure 2c,d). In this sense, mostly all of the particles are decorating the TiO_*x*_N_*y*_ island-like structures (Figure 3 and Figure S4). Overall, Pt nanoparticles are below 5 nm in size, with the majority of them around 2–3 nm (Figures S5 and S6). Commercial benchmark Pt/C TEC10E50E is supported solely on high-surface-area carbon, i.e., Ketjen black EC300 (BET = 800 m^2^ g^–1^) with the average size of Pt nanoparticles around 5 nm (Figures S5 and S6).

### 3.2 TF-RDE Comparison

**Figure 4:**
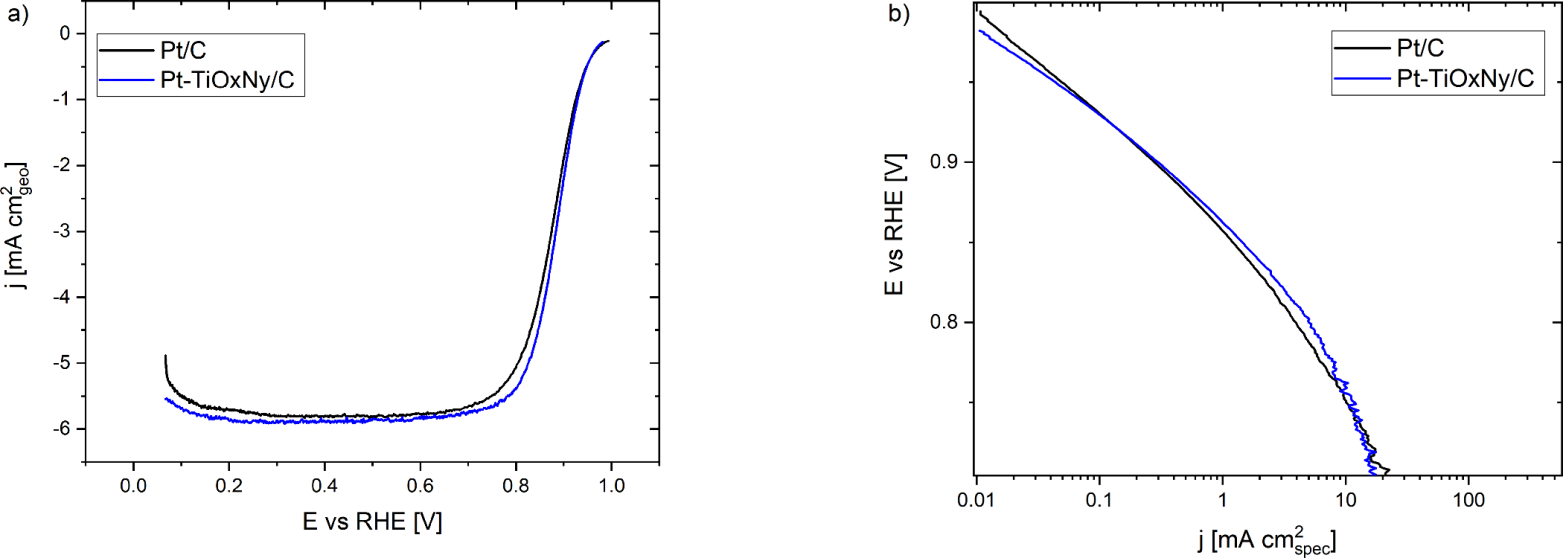
(a) TF-RDE comparison of the ORR polarization curves of the Pt-TiO_*x*_N_*y*_/C and Pt/C samples. (b) Tafel plots of the polarization curves normalized by the real Pt surface area. Both are recorded in 0.1 M perchloric acid with a potential sweep rate of 20 mV/s.

Initial electrochemical measurements targeted comparative analysis of the ORR performances of Pt/C and Pt-TiO_*x*_N_*y*_/C measured under a conventional TF-RDE configuration (Figure 4). Accordingly, at low overpotentials (at 0.9 V), Pt-TiO_*x*_N_*y*_/C demonstrates a comparable ORR-specific activity (ORR_spec_) within the error of measurement (Table 1). This is not in accordance with the findings on particle size-dependent ORR_spec_ reported in the past decades, which indicate a decreasing ORR_spec_ with a decreasing particle size. This trend is typically related to particles’ morphological changes resulting in alteration of the relative fraction of Pt surface atoms (edge sites vs facet sites). As the size decreases, the number of less active sites increases.^51−56^ Interestingly though, the obtained Tafel slopes in the Pt oxide region differentiate where values of 65 (Pt/C) and 56 mV/dec (Pt-TiO_*x*_N_*y*_/C) (Table 1) are obtained for the two analogues, respectively (Figure 4b). This is an anomaly that will be discussed later with regard to the Pt surface coverage (see section O/OH Adsorbate Coverage State Comparison). Rather surprisingly, in our case by decreasing the potential further (<0.9 V), the Pt-TiO_*x*_N_*y*_/C sample gradually outperforms the Pt/C analogue (Figure 4b). However, as mentioned previously, the uncertainty in calculating the kinetic current density out of RDE polarization curves increases with the ORR overpotential, i.e., when approaching the limiting current.^33^

**Table 1:** Structural and Electrochemical Characteristics of the Two Samples Investigated

catalyst	average particle size [TEM/nm]	Pt loading [wt %]	ORR_spec_ @ 0.9 V MFE [mA/cm^2^]	ORR_spec_ @ 0.9 V RDE [mA/cm^2^]	ECSA_CO_ [m^2^/g_Pt_]	ECSA_HUPD_ [m^2^/g_Pt_]
Pt/C	4.8 ± 1.5	50.6	0.12 ± 0.03	0.32 ± 0.09	48 ± 3	45 ± 5
Pt-TiO_*x*_N_*y*_/C	2.9 ± 0.8	18.4	0.0874 ± 0.0009	0.27 ± 0.05	66 ± 4	57 ± 8

### 3.3 MFE ORR Comparison of Pt/C vs Pt-TiO_*x*_N_*y*_/C

**Figure 5:**
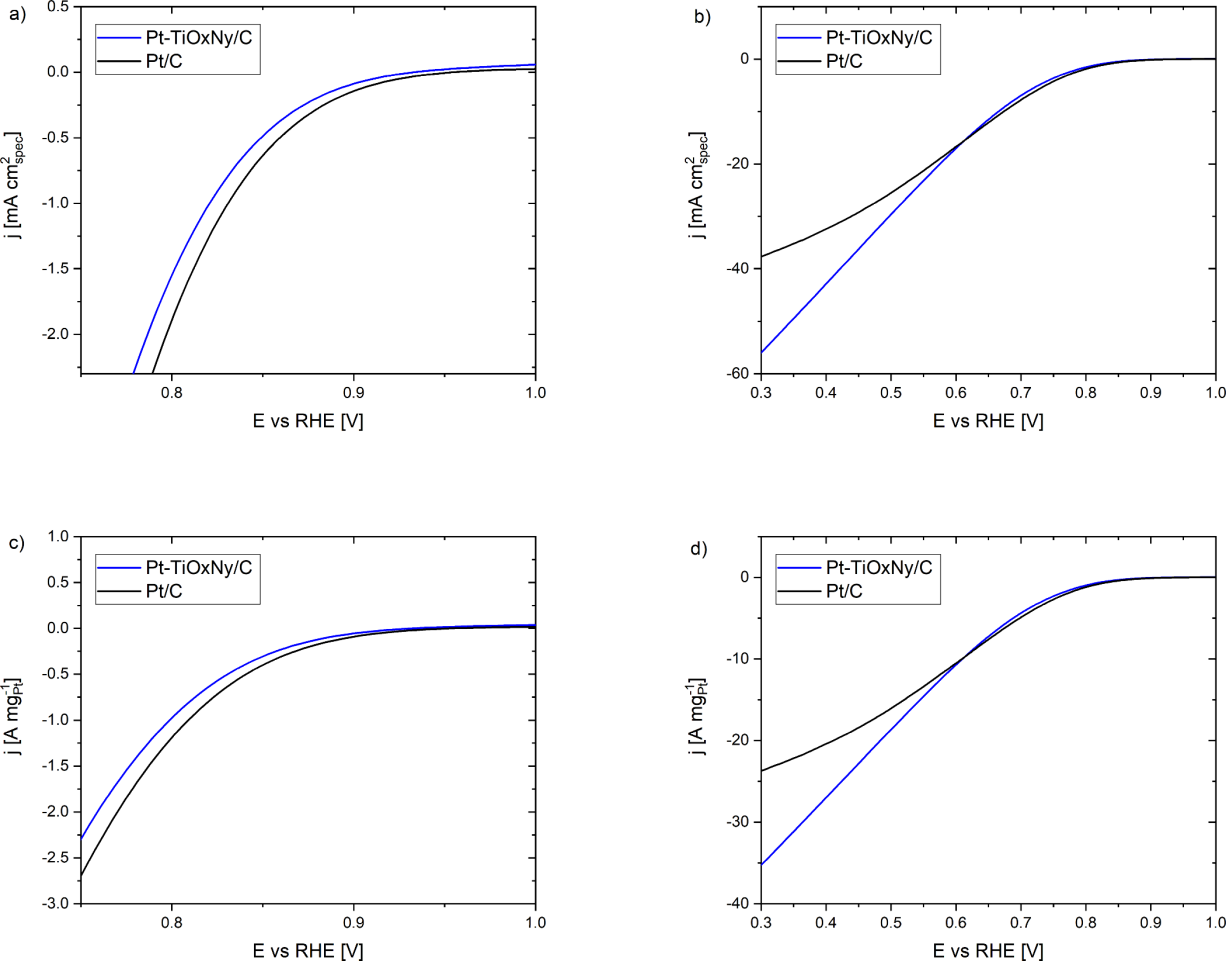
Comparison of the specific activity at (a) low overpotentials and (b) high overpotentials and mass activity at (c) low overpotentials and (d) high overpotentials of the Pt-TiO_*x*_N_*y*_/C and Pt/C samples, recorded in a 1 M perchloric acid electrolyte at the potential sweep rate of 20 mV/s.

Accordingly, our further investigation focused on exploiting the modified floating electrode setup the concept of which was adopted from the original idea of Kucernak’s group.^57^ The MFE design allows supplying gas-phase reactants directly to the working electrode (i.e., not through the liquid electrolyte) enabling fast mass transport and thus giving access to a wide potential window and significantly larger current densities in comparison to the RDE setup. Note that in contrast to our previous work,^49^ a 1 M HClO_4_ electrolyte instead of 4 M HClO_4_ was used in the present study to diminish the effect of ClO_4_^–^ blockage. For more details on MFE assembly, the reader is referred to the Experimental Section. Note that measures were taken to prevent the influence of the catalyst layer thickness on O_2_ mass transport (see SI Section S6). A direct comparison of the two analogues demonstrates that at low overpotentials (≥0.9 V), the samples demonstrate rather similar behavior (Figure 5a) closely following the TF-RDE trends (Figure 4b and Table 1). Interestingly, the ORR performance trend is substantially altered at high overpotentials, i.e., the Pt-TiO_*x*_N_*y*_/C significantly outperforms the Pt/C analogue (Figure 5b), a trend already suggested by the TF-RDE analysis (Figure 4b). Note, however, that due to MFE limitations (see Section S6) in the present comparative analysis, rather thin catalyst layers were used from the application perspective (<20 μg_Pt_/cm^2^_geom_), whereas larger loadings are typically used in the MEA configuration (down to ≈0.1 mg_Pt_/cm^2^). Nevertheless, the MFE trends at high overpotentials build on the confidence that under such conditions, the ORR performance is governed by intrinsic differences between the two analogues. This seems rather intriguing, especially if one considers particle size-dependent ORR_spec_, which has frequently been reported to increase with particle size.^51−56,58^ The most commonly accepted explanation is based on changes in the morphology with particle size, which is reflected in electrosorption properties. These tend to vary due to the change in the average coordination number leading to changes in particles’ ratios of crystal planes with less active sites becoming more abundant in smaller particles.^53,54,59^ Specifically, the population of undercoordinated sites (less ORR active) increases with decreasing the particle size in the range below 5 nm.^60−62^

Note again that the Pt particle size for the Pt-TiO_*x*_N_*y*_/C sample (2.9 nm) is smaller in comparison to the Pt/C analogue (4.8 nm), meaning that the expected particle size effect is not followed at the potentials below 0.9 V (Figure 5a,b). However, we emphasize that particle size-dependent ORR activity trends reported in the literature were predominantly obtained under the RDE configuration. Hence, knowledge beyond RDE-accessible potentials is scarce.^37,53^ Therefore, an explanation is needed to explain ORR_spec_ trends obtained at low potentials (<0.9 V) under the MFE regime. One possible explanation is that ORR_spec_ trends are affected by the interparticle separation distance. Namely, as proposed in a pioneered study by Watanabe et al., the distance between adjacent Pt particles rather than particle size is decisive for ORR activity in the case of supported Pt catalysts.^63^ Accordingly, the ORR_spec_ of a selected Pt particle is governed by the separation distance to neighboring particles. More specifically, if particles are sufficiently separated, O_2_ molecules are supplied by spherical diffusion to the individual particle.^64^ In the case of an insufficient particle-to-particle distance (<20 nm), diffusion field overlap results in decreased ORR_spec_. However, subsequent studies refuted the interparticle separation effect and instead correlated the ORR_spec_ to the surface morphology, which alters with particle size.^65,66^ Later studies also showed arguments against the influence of the interparticle distance. Namely, Arenz’s group demonstrated that ORR_spec_ in fact increases with decreasing the interparticle separation distance. Interestingly, the increase was not related to the surface morphology.^67,68^ The promotion effect is governed by the potential distribution in the electric double layer (EDL), i.e., the EDL overlapping effect, which alters the energetics of adsorbed blocking species (OH/O), hence increasing the ORR_spec_.

In regard to our result below 0.9 V (Figure 5), the following two facts need to be considered. First, the critical distance where the EDL overlap starts to significantly affect the ORR is below 1 nm,^67,68^ which cannot be said for the analogues investigated here as interparticle distances are on average notably larger (Figure 2). Second, the performance trends found in the literature were investigated in a narrow potential window (only above 0.8 V, i.e., very low current densities) limited by the RDE configuration employed. Hence, it remains elusive if the findings can be extrapolated to a high overpotential regime (high current density), i.e., well outside the region associated with higher coverages of oxide growth on the platinum surface. Therefore, we recognize the recent work by Kucernak’s group as the most credible study to rely upon regarding the ORR trends at high overpotentials.^37^ In this case, the potential window of interest was expanded to 0.3 V by implementing the original floating electrode technique (FET). Importantly, their work systematically investigated Pt/C analogues with fixed platinum-to-carbon ratios on a variety of Pt particle sizes. This is of significant relevance as under such conditions, the interparticle distance decreases with particle size. According to above-mentioned Watanabe’s rationale,^63^ this should result in higher ORR_spec_ for larger particles (increase of interparticle distances), whereas the ORR_spec_ should increase with a particle size decrease according to Arenz’s rationale (the interparticle distance should decrease). FET measurements disclosed that ORR_spec_ increases with particle size in a wide potential range.^37^ Similarly, we included an additional Pt/C analogue in comparative analysis, namely, a sample from the same producer as the reference Pt/C (4.8 nm), but with a distinctly smaller average particle size (2.6 nm). Note that the obtained ORR_spec_ trend follows the ones from FET, i.e., larger particles being more active (Figure S7). We emphasize that direct comparison between the FET and MFE is not possible, as the two setups have distinctive differences in terms of the electrode architecture. In fact, achieving the same ORR_spec_ under the MFE regime compared to FET is likely impossible. Specifically, FET utilizes a more hydrophobic character and positions the catalyst within well-defined pores of the electrode substrate, creating an array-like catalyst layer. This combination enables ORR proceeding with (probably entirely) diminished mass transport limitations; hence, higher current densities are reached under FET.^57^ This is in contrast to the MFE, where the catalyst layer is spread throughout the entire grid, including the continuous catalyst layer in the holes of the holey carbon. As a result, the catalyst layer in the MFE is more flooded, leading to mass transport limitations. Therefore, the MFE can be used to compare two different catalysts but in a relative manner. Regardless, since both techniques give the same trend for the particle size effect (for Pt/C analogues), this indicates that neither the EDL overlapping effect nor the interparticle distance seems to play a role at low potentials. Specifically, in Kucernak’s study, the performance increase with particle size was related to changes in the site ratio (edges and facets), which was further supported by the electrokinetic model. Based on fitting the polarization curves^36^ incorporating site blockage of O, OH, OOH, and H, the authors demonstrated that O_2_ adsorption becomes the rate-limiting step at high overpotentials. Accordingly, larger particles (with a larger fraction of facets) are more active. It seems, therefore, that in our particular instance, the ORR_spec_ performance difference at high overpotentials is not related to particle size effects as the analogue with smaller particles (Pt-TiO_*x*_N_*y*_/C) is more active (Figure 5b) in contrast to the trend within Pt/C analogues (Figure S7). Obviously, in order to completely refute the relevance of the interparticle distance, one would need to synthesize a library of analogues on both types of supports with well-defined distances. This might pose a great challenge in terms of synthesis, and the analogues would only be relevant for MEA testing. Specifically, the interparticle distance becomes of significant importance under the high current density regime (>1 A cm^–2^) where the local O_2_ resistance at the Pt/ionomer interface (*R*_O2_^Pt^) plays a pivotal role. However, *R*_O2_^Pt^ increases with the interparticle distance leading to lower ORR performance,^38^ i.e., a trend in contrast with Watanabe’s rationale. We also note that it is unlikely that *R*^*Pt*^_O2_ were to limit the reaction for the cases of Pt-TiO_*x*_N_*y*_/C and Pt/C, as such a limitation should be the same for both analogues. This would then result in the convergence of ORR_spec_ at low potentials. Note that this is not the case (Figure 5b).

### 3.4 CO Stripping Probe

**Figure 6:**
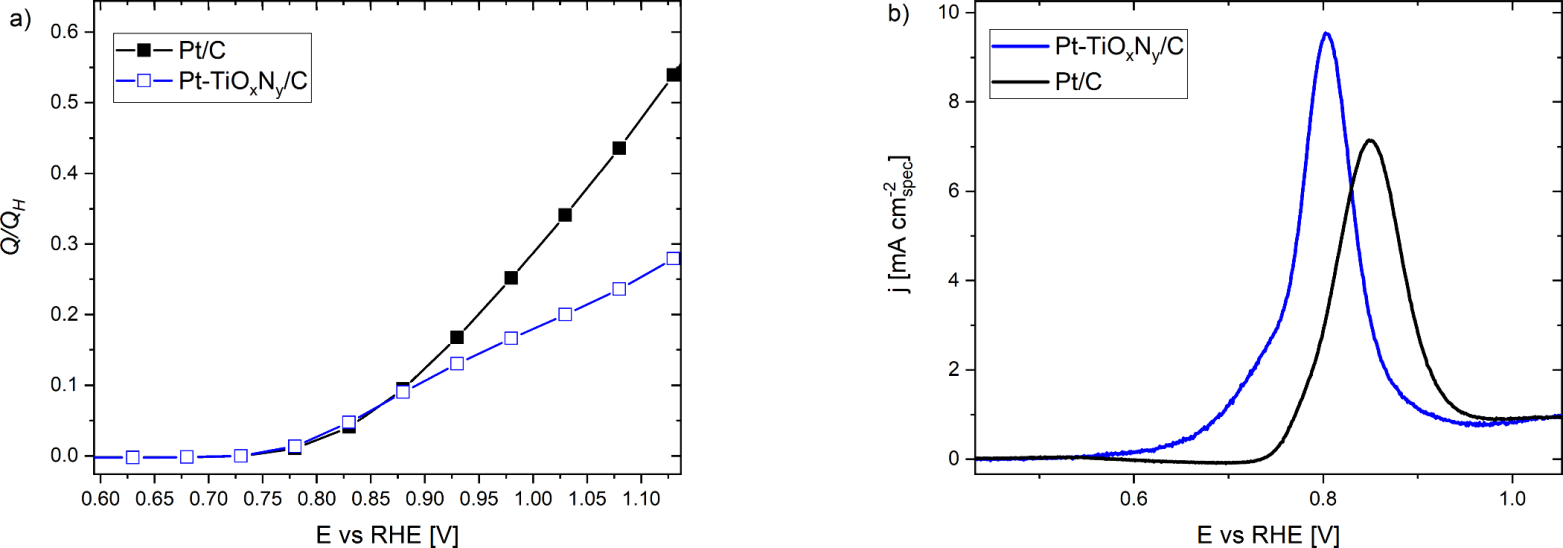
(a) Apparent surface coverage of oxygen species on the Pt surface calculated from CVs in an Ar atmosphere using the methodology of Pasti et al.^47^ (b) Comparison of the CO stripping peaks of Pt-TiO_*x*_N_*y*_/C and Pt/C; curves normalized in terms of the specific surface area (cm^2^_real_) using the charge under CO_stripp_ and corrected for the background current from the double-layer capacitance. Both measurements are recorded with a potential sweep rate of 50 mV/s in 0.1 M perchloric acid.

Nevertheless, our further investigation focused on obtaining more information regarding the surface properties of the two analogues. Accordingly, we compared the voltammetric response of CO monolayer oxidation (the so-called CO stripping, CO_stripp_), which is strongly susceptible to alterations in the Pt surface.^69^ The reaction proceeds via the Langmuir–Hinshelwood-type mechanism where CO_ad_ and the oxidizing agent OH_ad_ have to be coadsorbed on the adjacent sites at the same time.^70^ As demonstrated before, the CO_stripp_ peak shifts to positive potentials with decreasing the Pt particle size.^71^ This was explained by kinetic modeling, which suggested the particle size-dependent diffusion coefficient causing a restricted surface mobility of CO_ads_ with a decreasing particle size.^71,72^ OH_ad_ on the other hand is considered as immobile^73^ and exclusively located on the active sites. These are to be related to defects^74,75^ toward which CO_ad_ is supplied by surface diffusion. From the comparative analysis, it is evident that the two analogues demonstrate anomalous behavior (Figure 6b), and instead, the CO_stripp_ peak is shifted cathodically in the analogue with smaller particles (Pt-TiO_*x*_N_*y*_/C). This indicates that the obtained CO_stripp_ voltammograms (Figure 6b) cannot be explained by the CO_ad_ surface mobility, as this process is more restricted for the smaller particles. Instead, the trend implies either a higher fraction of surface defects for the Pt-TiO_*x*_N_*y*_/C analogue or an OH supply by TiO_*x*_N_*y*_ via spillover as suggested by the literature.^76−81^ We argue that the spillover mechanism seems more likely than the influence of defects since in the latter, the surface coverage trend should disclose higher coverage for the Pt-TiO_*x*_N_*y*_/C analogue (see the following section).

### 3.5 O/OH Adsorbate Coverage State Comparison

To obtain more insight into the surface coverage, we conducted a voltammetric analysis. Briefly, the determination of Pt surface coverage with oxygenated adsorbates, i.e., hydroxyl/oxide species, is based on constructing the apparent surface coverage (θ) obtained under different scan rates (see the Experimental Section for a detailed description). By systematically reducing the sweep rate, more OH/O species are allowed to accumulate on the surface in the course of an anodic scan, which is in agreement with hydroxide/oxide formation on the Pt surface.^46,82^ Note that this voltage scan rate trend is valid for both analogues investigated here where the surface coverage increases with an increase in potential and a decrease in the sweep rate (see SI Section S7).^83^ However, a comparative analysis of the two analogues discloses that the Pt-TiO_*x*_N_*y*_/C sample manifests smaller surface coverage from 0.9 V onward indicating a weaker interaction with OH/O species (Figure 6a and SI Section S7). This is in contrast with the smaller slope obtained for the Pt-TiO_*x*_N_*y*_/C analogue (Figure 4b), as a decrease in the OH_ad_ coverage should typically result in a larger Tafel slope.^35,84,85^ Instead, the slope anomaly should be ascribed to partial encapsulation of Pt particles with a thin layer of TiO_*x*_N_*y*_ origin resolved via IL-TEM analysis (see section Identical Location Scanning Transmission Microscopy (IL-STEM)), which blocks a fraction of the Pt surface. The surface coverage trend is an anomaly if considered from the perspective of the particle size effect. Namely, as demonstrated for Pt/C analogues with decreasing the particle size, the oxophilicity of the surface increases, i.e., the smaller particles are more irreversibly oxidized at lower potentials than larger ones.^54,62^ Specifically, the population of undercoordinated sites increases with decreasing the particle size, in particular in the range below 5 nm.^60−62^ The alteration of the adsorption properties results in stronger OH_ad_ bonding for smaller particles.^86^ Note again that the Pt particle size for the Pt-TiO_*x*_N_*y*_/C sample (2.9 nm) is smaller in comparison to the Pt/C analogue (4.8 nm); hence, one would expect higher surface coverage for the former, which is not the case here (Figure 6a). The tendency toward oxidation would play a pivotal role in electrochemical stability,^87,88^ which is planned for our future study. In terms of ORR activity, the coverage with oxygenated adsorbates should reversely scale with activity. Interestingly, this is in contrast to our results, namely, the activity in the potential window of lower coverage (>0.9 V, Pt-TiO_*x*_N_*y*_/C) is lower (Figure 5a). This as well should be directly related to partial blockage of the Pt surface (see section Identical Location Scanning Transmission Microscopy (IL-STEM)). Note that the activity at low potentials (i.e., the same apparent surface coverage) is higher for the Pt-TiO_*x*_N_*y*_/C analogue (Figure 5a). In this potential window, the Pt surface is free of adsorbed OH/O blocking species; therefore, their influence should be excluded. This isolates the reaction intermediates (e.g., H_ad_ and HOO_ad_) as the only surface species, which could influence ORR proceeding, which are not considered in the surface coverage analysis. Therefore, we hypothesize that other Pt–support interactions are at play at low potentials.

### 3.6 Identical Location Scanning Transmission Microscopy (IL-STEM)

**Figure 7:**
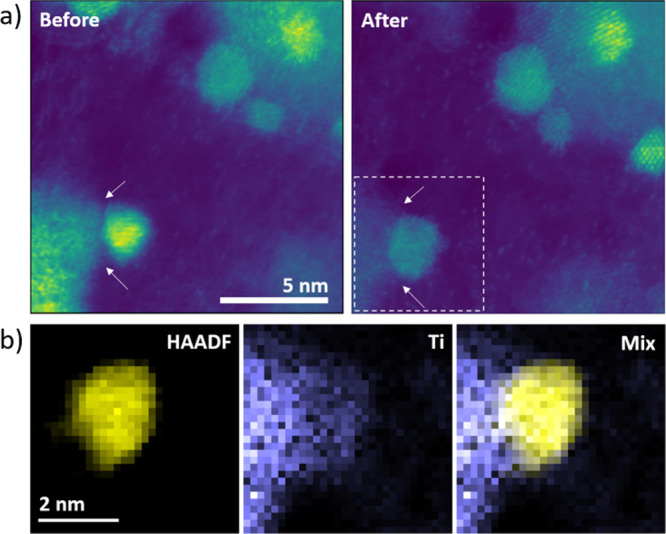
(a) Before and after the IL-4DSTEM ADF. A small gap exists initially between the particle and the support, which disappears afterward. The change in contrast that we observe in the particle may be due to diffraction contrast after a slight change in orientation. (b) HAADF and EELS Ti signals from the dashed-marked region. The Pt nanoparticle sits in a pocket-like juncture support.

Since recent studies on oxide-based composites disclosed that Pt might be subjected to surface poisoning due to the growth of a thin oxide layer, our further efforts pursued this phenomenon. The reported layers originate from an oxide-based support and can form during composite synthesis or electrochemical perturbation.^89−91^ This might cause at least partial encapsulation of the Pt nanoparticles. Note that metals having relatively high surface energy (such as Pt and Pd) are prone to encapsulation.^92^ The resulting layers were reported to be permeable for H^+^ (H_2_) and impermeable for O_2_, H_2_O, and CO.^89−91^ This means that the fraction of the effective Pt area for the ORR is lower for the case of Pt-TiO_*x*_N_*y*_/C if indeed some encapsulation layer is present. Note that the surface coverage analysis implies the presence of partial surface blockage as lower coverage is obtained for the Pt-TiO_*x*_N_*y*_/C analogue (Figure 6a). Furthermore, the encapsulation could explain the discrepancy between the surface coverage analysis and the ORR_spec_ performance at low overpotentials (Figure 5a). To indirectly observe the presence of partial encapsulation, HOR was exploited as a surface probe. Specifically, HOR performance typically rapidly declines with the initiation of the Pt oxide region due to the surface blockage. However, from the comparative analysis, it is evident that the Pt-TiO_*x*_N_*y*_/C analogue outperforms Pt/C at high potentials, indicating that the Pt surface is predominantly nonencapsulated; however, a fraction of the Pt surface is indeed less covered with oxygenated adsorbates in Pt-TiO_*x*_N_*y*_/C (see Figure S13). To clarify whether the coverage of Pt with the (at this point still hypothetical) layer increases due to electrochemical biasing, we performed accelerated stress tests (AST) for the Pt-TiO_*x*_N_*y*_/C analogue in order to pursue the ORR performance trend and CO_stripp_ response before and after the AST protocol (see Section S8). Note that the increase of encapsulation should manifest in ORR polarization as well as in CO_stripp_ response as both reactions should be inhibited due to impeded transport of reactants.^89−91^ According to our results, this is evidently not the case as both reactions show an unaltered performance (Figure S12). Finaly, to unambiguously address the phenomenon of the encapsulation layer, we performed a dedicated TEM analysis of Pt-TiO_*x*_N_*y*_/C before and after electrochemical perturbation (EP, 300 cycles, 0.05–1.2 V, 300 mV/s) under the identical location mode (the so-called IL-TEM approach). In particular, IL-STEM was carried out. After imaging several regions, the most obvious feature is Pt single atoms (SA), which are present; however, they are present also prior to EP (Figure S14). We note that the SA contribution toward ORR is negligible, which was verified by measuring the ORR of Pt-TiO_*x*_N_*y*_/C composed of Pt SAs solely (data not shown). In addition, some regions of the TiO_*x*_N_*y*_ support suffered from dissolution, while others appeared to show redeposition (Figure S15). Although this may be a common phenomenon, it would usually be unnoticed since it is hard to observe it if an identical location is not conducted. In that sense, the likely overlayer growth was tracked on Pt nanoparticles that were located at the edge of the TiO_*x*_N_*y*_ support. In Figure 7a, IL-4DSTEM ADF images show that before the EP protocol, there is a small gap between a Pt nanoparticle and the TiO_*x*_N_*y*_ support. After the EP protocol, the Pt nanoparticle and the TiO_*x*_N_*y*_ support appear to be joined in a more profusely manner since the small gap between the two has faded away. Yet, due to the change in contrast in the particle, a slight tilt may be occurring and hence giving a different perspective. In order to confirm the layer formation at the junction between the Pt and TiO_*x*_N_*y*_ support, electron energy loss spectroscopy (EELS) was employed to map the signal of Ti. In Figure 7b, the Ti signal is overlaid with the HAADF signal that mostly arises from Pt due to *Z*-contrast. By looking at the Ti map, it is evident that there is some signal at the position of the Pt particle. This can be pictured as the Pt nanoparticle sitting on the TiO_*x*_N_*y*_ support in a pocket-like fashion. Other regions with similar characteristics were analyzed, displaying similar behavior.

### 3.7 Strain Analysis

**Figure 8:**
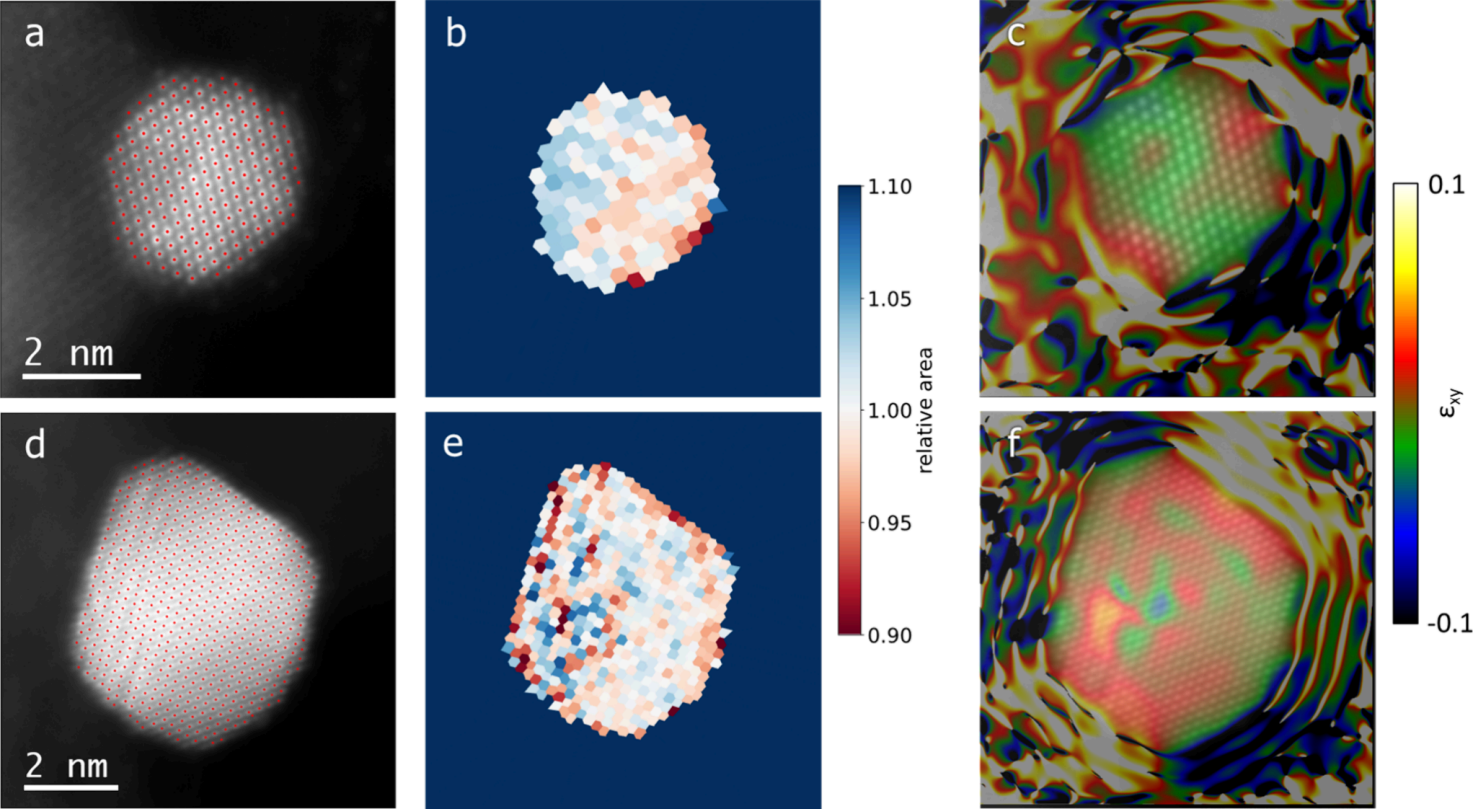
Strain analysis of TiO_*x*_N_*y*_-C- (top) and C-supported (bottom) Pt nanoparticles. (a,d) HAADF-STEM images of Pt-TiO_*x*_N_*y*_-C and Pt–C nanoparticles with overlaid atomic column positions in red. (b,e) Voronoi diagrams for both nanoparticles, with colors corresponding to relative unit cell areas. (c,f) Shear strain ε_*xy*_ maps for both nanoparticles.

To obtain more information about the Pt–support interaction, we conducted a more detailed STEM investigation, focusing on possible differences in Pt nanocrystal strain effects. The experimental approach is based on analyzing HAADF-STEM micrographs of Pt particles located at the periphery of the supporting material, TiO_*x*_N_*y*_/C or carbon, suggesting partial support. Figure 8a,d exhibits STEM images of Pt-TiO_*x*_N_*y*_-C and Pt–C nanoparticles with atomic column positions marked with red dots. The latter was used to construct a Voronoi diagram, as shown in Figure 8b,e. In a Voronoi diagram, an image is split into multiple cells, in which each cell contains exactly one atomic column. Cells consist of all points of the image that are closer to this particular atomic column than to any other. Thus, they were used to measure the unit cell areas around each atomic column relative to the average cell area of each respective nanoparticle. The resulting images of both nanoparticles, imaged in a [110] zone axis at the edge of their respective supports, provide visual evidence of how the crystal structure of the supported part of a nanoparticle can be altered by epitaxial growth for a certain choice of support material. From the Voronoi analysis (Figure 8b), it is evident that there is a difference in the interatomic distance between the supported and unsupported parts of the Pt particle in the case of the Pt-TiO_*x*_N_*y*_/C analogue with the supported part exhibiting larger cell areas compared to the unsupported one. In the case of the Pt/C analogue (Figure 8e), there is no similar trend when comparing the overall cell areas from both parts of the nanoparticle. However, there are some local fluctuations in cell areas in the supported part, which can be ascribed to internal defects, as apparent also from the original HAADF-STEM image. Additionally, geometric phase analysis (GPA) was conducted to confirm any strain effects, as shown in Figure 8c,f. The shear strain results for the Pt-TiO_*x*_N_*y*_/C particle (Figure 8c) indicate that there is some strain accumulation in the middle of the particle, approximately where one could expect the outer border of the support, roughly agreeing with the domains exhibiting lower and higher relative unit cell areas, as depicted in Figure 8b. The strain map of the Pt/C nanoparticle demonstrates some strain accumulation in regions, exhibiting defects, but no systematic differences in the unit cell parameter between various parts of the nanoparticle, again agreeing with the Voronoi analysis (Figure 8f). However, despite the noticeable trend in this chosen imaging projection, differences between the supported and unsupported regions of nanoparticles might manifest themselves differently in other projections. Overall, the structural analysis of the Pt-TiO_*x*_N_*y*_/C analogue, as revealed by HAADF-STEM images and the subsequent strain analysis, provides solid evidence of the platinum–support interaction. Namely, as the interatomic distances between the supported and unsupported parts of the Pt particle notably differ (i.e., causing strain), the platinum–support interaction in the Pt-TiO_*x*_N_*y*_/C analogue is most likely an effect of the local atomic arrangement of the Pt nanoparticles.^93^ This should in principle have direct consequences on electrocatalytic properties and thus ORR. Namely, the platinum crystal strain was shown to shift the electronic d-band structure leading to altered chemisorption of oxygenated species such as OH and O, i.e., ORR site-blocking species at low overpotentials, and has firmly established itself as an ORR promoter.^9,94,95^ Provisionally, the strain analysis closely agrees with the voltammetric Pt surface coverage trends (Figure 6a). However, according to the ORR trends at high potentials, the strain likely does not play the predominant role (Figure 5a). Instead, the partial blockage of the Pt surface with the encapsulation layer (see section Identical Location Scanning Transmission Microscopy (IL-STEM)) inhibits the activity for the Pt-TiO_*x*_N_*y*_/C analogue. However, when considering the high current density trends (low potentials, Figure 5a,b), the strain seems to lower the adsorption of ORR intermediates (i.e., HOO_ad_), which are relevant spectators under low potentials.^36^ Alternatively, the change of the ORR mechanism at low potentials could as well lead to obtained ORR trends; however, according to the recent high current density regime study based on FET and kinetic modeling, this seems highly unlikely.^37^

## 4 Conclusions

In the present study, we provide a comparative analysis of carbon (Pt/C) and titanium oxynitride support-based Pt particles (Pt-TiO_*x*_N_*y*_/C) predominantly focusing on ORR performance. For this purpose, we employ a modified floating electrode (MFE) to assess the reaction across a wide, PEMFC-relevant potential window. The investigation under high mass transport, that is, at high current density, demonstrates enhanced activity for the Pt-TiO_*x*_N_*y*_/C catalyst when compared to the Pt/C industry benchmark catalyst. The 3-times activity increase in the high current density is attributed to a lower platinum surface coverage with ORR intermediates, which leads to higher availability of active sites for O_2_ adsorption. The altered ORR characteristics show anomalous behavior in terms of the particle size effect, which we ascribe to the following two effects. First, the influence of electronic Pt–support interaction induces strain within the Pt nanoparticles’ crystal structure in the Pt-TiO_*x*_N_*y*_/C analogue according to the HAADF-STEM strain analysis. Second, Pt particles are partially encapsulated within a thin layer of TiO_*x*_N_*y*_ origin, which is supported by several electrochemical surface probe approaches, namely, voltammetry-based surface coverage analysis and HOR probing. Both imply that Pt in the TiO_*x*_N_*y*_/C analogue is more resilient toward surface/subsurface oxidation. Finally, the partial encapsulation is directly disclosed by a detailed IL-TEM characterization with advanced approaches such as 4DSTEM acquisition. Overall, TiO_*x*_N_*y*_/C-based supports in conjunction with platinum nanoparticles seem to be a promising strategy to lower the Pt loading and increase the ECSA without sacrificing the catalytic activity and, based on preliminary testing, also stability under fuel cell-relevant potentials.

## Supporting Information Available

Details on the Pt-TiO_*x*_N_*y*_/C synthesis, XRD characterization, scanning transmission electron microscopy characterization, MFE measurements of Pt/C analogues, “break-in” protocol description, CO stripping simulation protocol description, loading-independent region in MFE measurements, adsorbate coverage state comparison, accelerated stress test measurements, hydrogen oxidation reaction measurements, and identical location microscopy characterization (PDF)
