# Supplementary material for: Metal–Support Interaction between Titanium Oxynitride and Pt Nanoparticles Enables Efficient Low-Pt-Loaded High-Performance Electrodes at Relevant Oxygen Reduction Reaction Current Densities
Source: ACS Catal. 2024 Feb 2;14(4):2473–86. doi: 10.1021/acscatal.3c03883 (PMC10877567; doi:10.1021/acscatal.3c03883)
Supplement: Supplementary file 1 — cs3c03883_si_001.pdf [file cs3c03883_si_001.pdf]

Metal Support Interaction between Titanium Oxynitride and Pt Nanoparticles Enables Efficient Low-Pt-Loaded High-Performance at Relevant Oxygen Reduction Reaction Current Densities

Armin Hrnjić[a,b,*], Ana Rebeka Kamšek[a,c], Lazar Bjelić[a,b], Anja Logar[a,b], Nik Maselj[a,c], Milutin Smiljanić[a], Jan Trputec[a,c], Natan Vovk[a,c], Luka Pavko[a,c], Francisco Ruiz-Zepeda[a], Marjan Bele[a], Primož Jovanovič[a,*], Nejc Hodnik[a,b,*]

[a] *Department of Materials Chemistry, National Institute of Chemistry, Hajdrihova 19, 1000, Ljubljana, Slovenia*

[b] *University of Nova Gorica, Vipavska 13, 5000, Nova Gorica, Slovenia*

[c] *Faculty of Chemistry and Chemical Engineering, University of Ljubljana, Večna pot 113, 1000, Ljubljana, Slovenia*

Corresponding authors:
armin.hrnjic@ki.si
primoz.jovanovic@ki.si
nejc.hodnik@ki.si

Supporting Information

S1. Pt-TiO_xN_y/C synthesis

The synthesis procedure is the same as described in our recent publication.[1] In the first step graphene oxide nanoribbons (GONR) were prepared.[2] 8 g of C-grade MWCNT (NTL) were added into a mixture of 1000 ml of sulfuric acid (Carlo Erba, 96%) and 110 ml of phosphoric acid (Merck, 85%) and stirred. Over the next four days, 8 wt. eq. of $KMnO_4$ (8 x 8 g) was added to the mixture under stirring. The mixture was then quenched with ice, followed by adding 30 % H_2O_2 until the colour changed from purple to yellowish. The supernatant was discarded, a portion of ultrapure water (resistivity 18.2 MΩ cm, obtained from Milli-Q Direct Water Purification System, MilliPore) was added, and the mixture was centrifuged for 30 minutes at 10500 rpm (Sorvall LYNX 4000, Thermo Scientific). Obtained solid was re-dispersed in 5 % HCl for 2 hours to eliminate any residual metals. Afterwards, the mixture was centrifuged for 30 minutes at 10500 rpm, followed by the supernatant decantation. The last cleaning step comprised of re-dispersing the GONR in ultrapure water and soaking it until the next day, followed

by centrifugation at 10 500 rpm for 1 h to discard the supernatant. A total of 5 washing cycles in ultrapure water were conducted. Afterwards, GONR were re-dispersed in ultrapure water with a concentration of ~ 20 g/L and treated with the ultrasonic bath (Iskra Sonis 4, Iskra) for 15 min to exfoliate the product. The suspension was then freeze-dried to obtain the dry product. In the next step, TiO_2 coating on GONR was prepared. For this purpose, 0.1 g of dried GONR was mixed with 1 mL of propanol (Honeywell, 99.8%) solution containing 0.5 mmol of Ti isopropoxide (Aldrich, 97%). After mixing at room temperature, Ti isopropoxide was hydrolysed by adding 0.2 mL of water (Milli-Q water, 18.2 MΩ cm). The obtained mixture was then dried in air at 50°C. In the third step, a water solution containing 35 mg of $Pt(NH_3)_4(NO_3)_2$ (Alfa Aesar) (1 mL) was added to the dried mixture and lightly milled in a mortar at 50°C until evaporation. Afterwards, the mixture was thermally treated in a 90% NH_3, 9.5% Ar, and 0.5% H_2 mixture. The temperature was first increased at a rate of 2°C min^{-1} to 250°C for 2 h, then at a rate of 10°C min^{-1} to 730°C for 3 h, and then cooled to room temperature with a rate of 10°C min^{-1}. The final Pt-TiO_xN_y/C material contained 18.4 wt. % of Pt, according to the ICP-OES analysis.[1] A commercial Pt/C analogue (TEC10E50E-HT, TKK, Japan) was selected for comparison and contained 50.6 wt. % of Pt.

S.2 Structural characterization

S2.1 X-ray diffraction

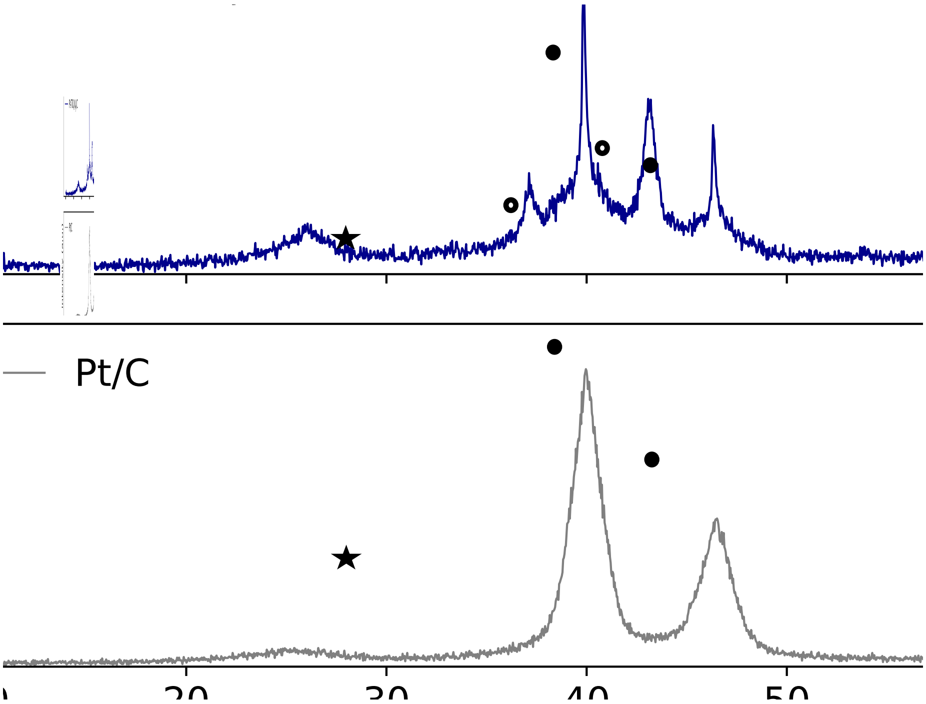

Figure S1: X-ray diffraction patterns of Pt-TiO_xN_y/C (top) and Pt/C (bottom). Empty circular markers denote the TiO_xN_y phase, filled circular markers denote the Pt phase, and the star marker denotes carbon.

S2.2 Scanning transmission electron microscopy

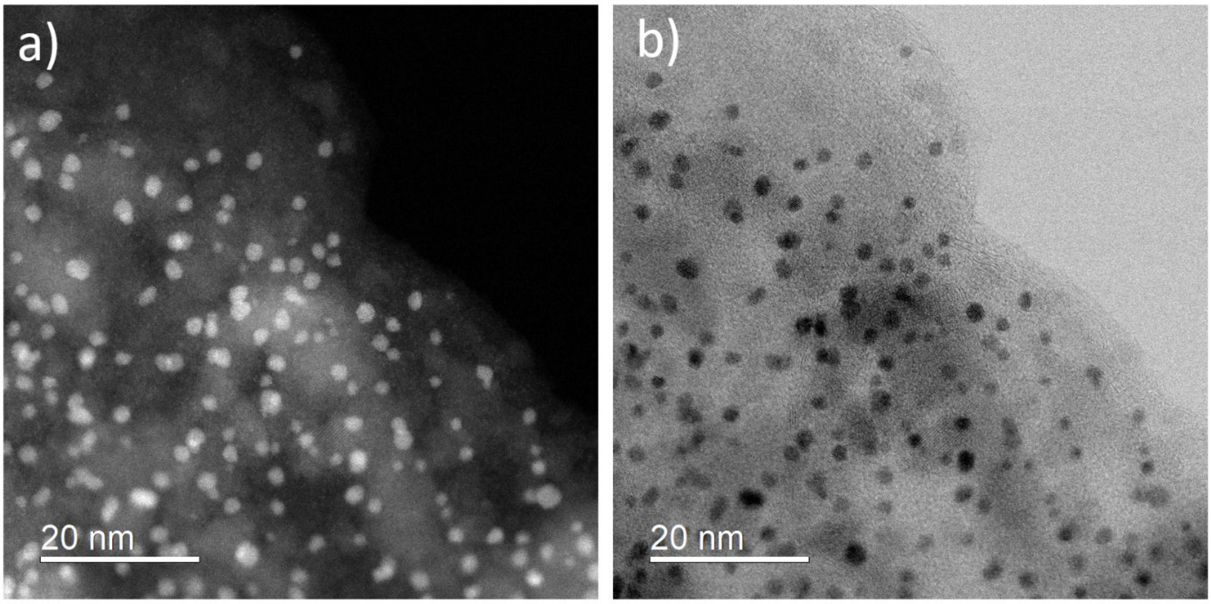

Figure S2: HAADF-STEM images of the Pt-TiO_xN_y/C sample at different magnifications showing the structure and morphology of the sample.

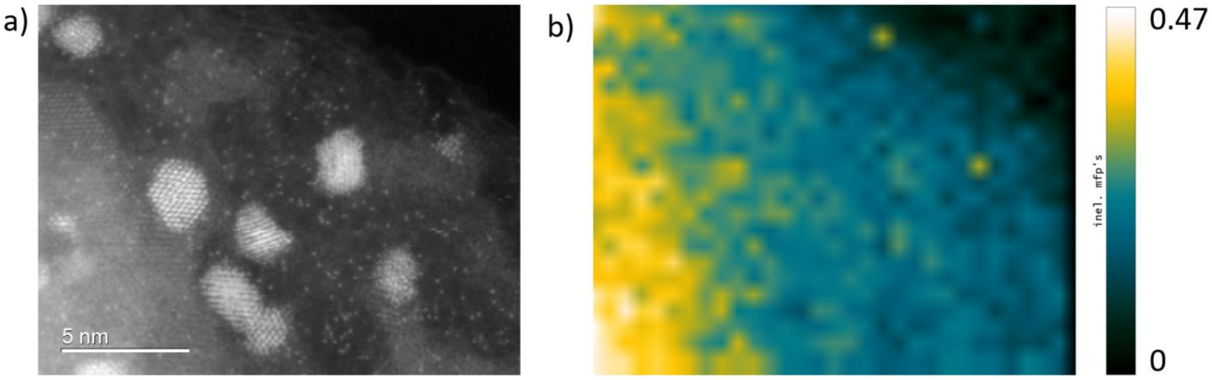

Figure S3: a) STEM-HAADF and **b)** EELS thickness map. Although both TiO_xN_y and C thicknesses vary across the sample, in this case a gradient can be seen in the map. This allowed us to estimate roughly the thicknesses of the TiO_xN_y islands-like structures ranging between 3 nm to 10 nm. The scale corresponds to t/λ. According to the microscope conditions employed, the calculated MFP (mean free path, λ) for C is ~72.64 and for TiON is ~58.3. From the EELS signal, the estimated average O:N ratio of some of the TiO_xN_y structures yielded 1.78:1, corresponding to $TiO_{1.28}N_{0.72}$.

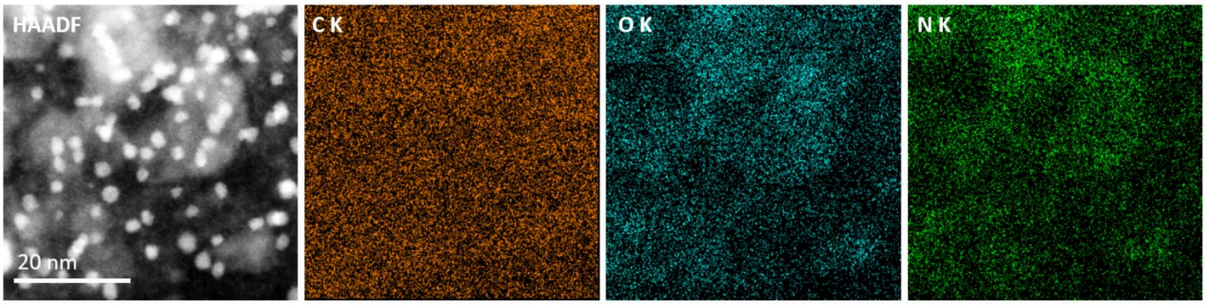

Figure S4: STEM-HAADF and EDXS signal maps corresponding to C K, O K, and N K complementing **Figure 3** in the main text.

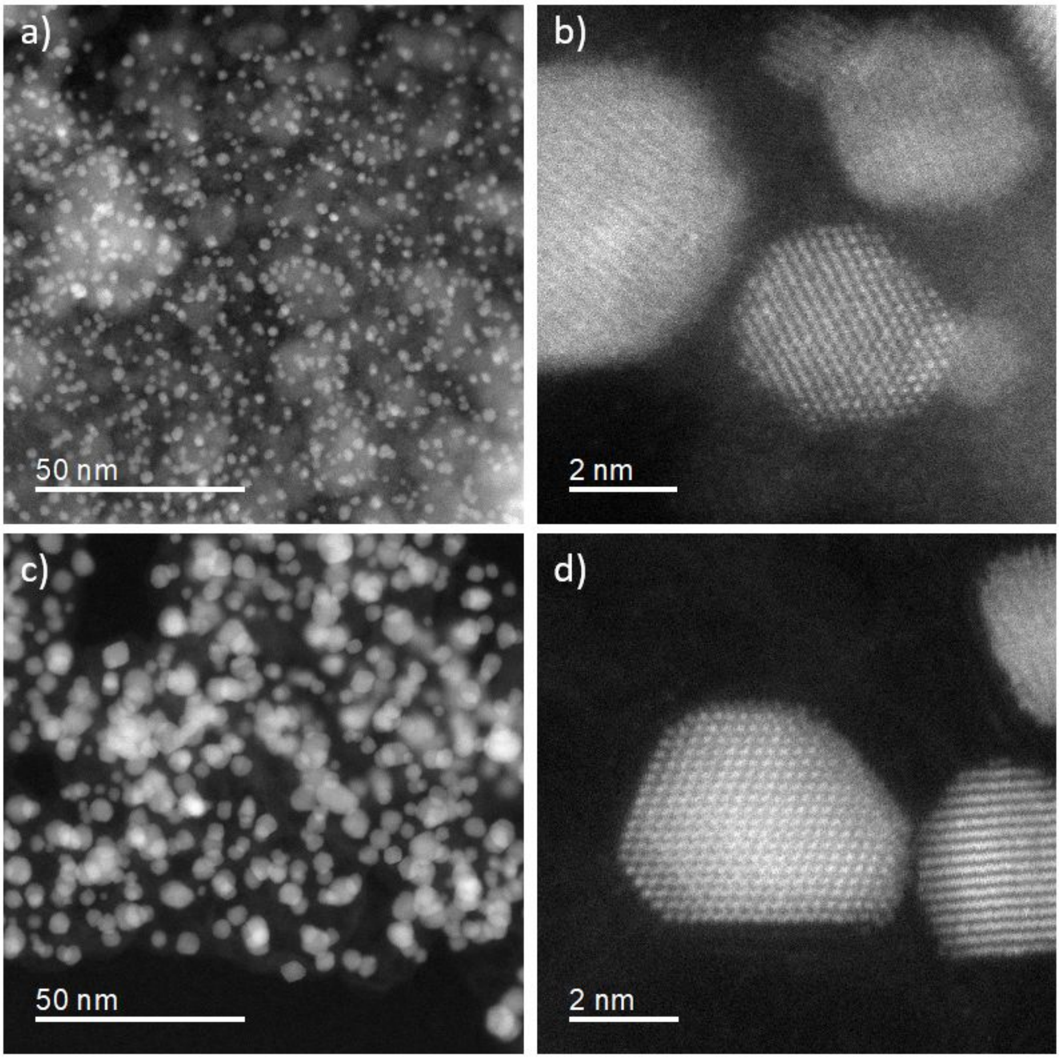

Figure S5: STEM-HAADF images of **a)** & **b)** Pt-TiO_xN_y/C and **c)** & **d)** Pt/C. Notice the difference in size between Pt nanoparticles in both samples.

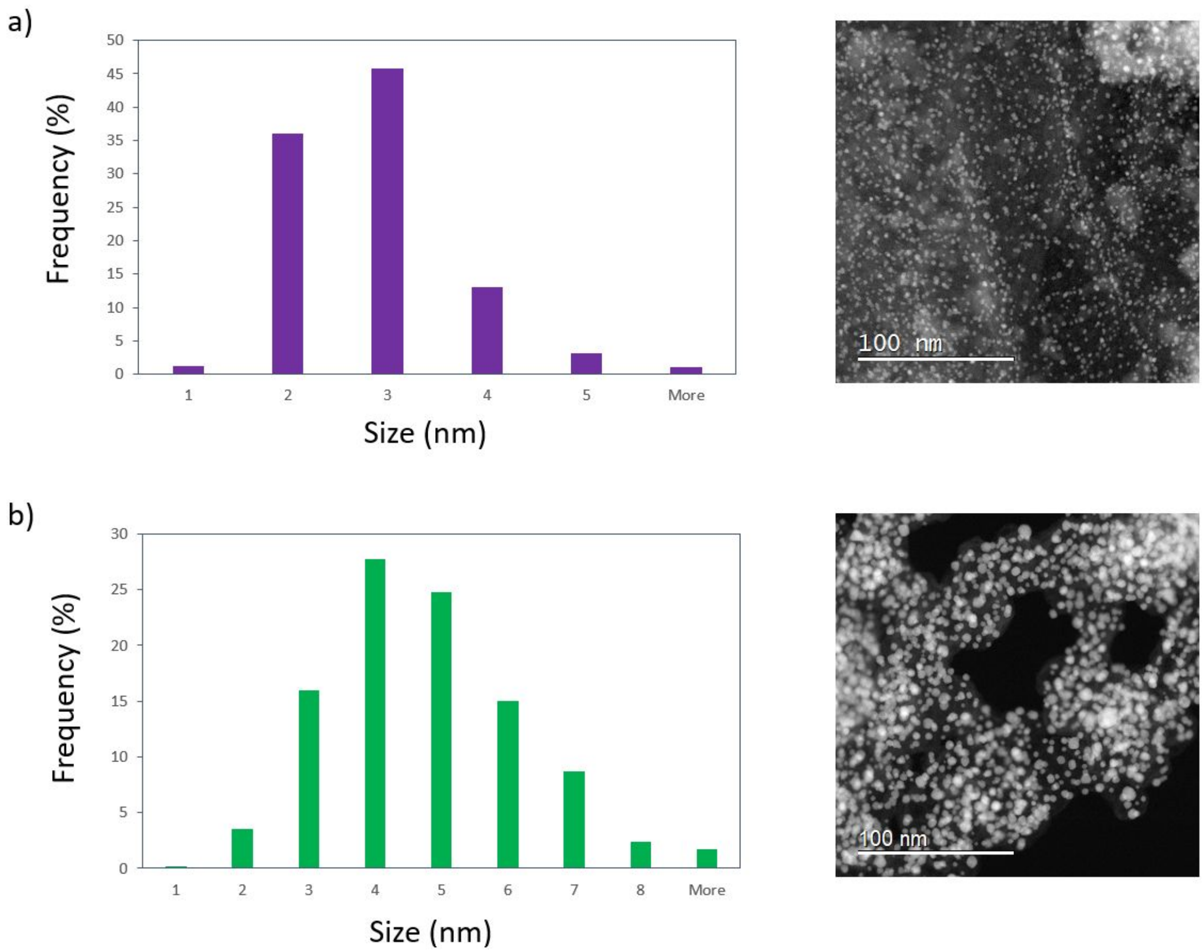

Figure S6: Particle size distribution charts and STEM-HAADF images of **a)** Pt-TiO_xN_y/C and **b)** Pt/C samples.

S3. "Break-in" protocol in MFE measurements

The following voltammetric pre-treatment was performed prior to ORR performance assessment with MFE that was adapted from Lin et. Al.[3] Our break-in protocol consisted of performing CVs in a hydrogen and oxygen atmosphere for 7 cycles (**Scheme 1**). The break-in protocol is important to establish consistent and reproducible results and to achieve optimal performance of the electrode. It helps generate the proton pathways and gas channels in the ionomers and the hydrophobic coating within the catalyst layer required for efficient catalyst performance. The break-in procedure involves performing successive ORR and HOR reactions on the catalyst with the lower potential limit extending to −0.1 V to create proton pathways and remove residual poisons on the catalyst surface.

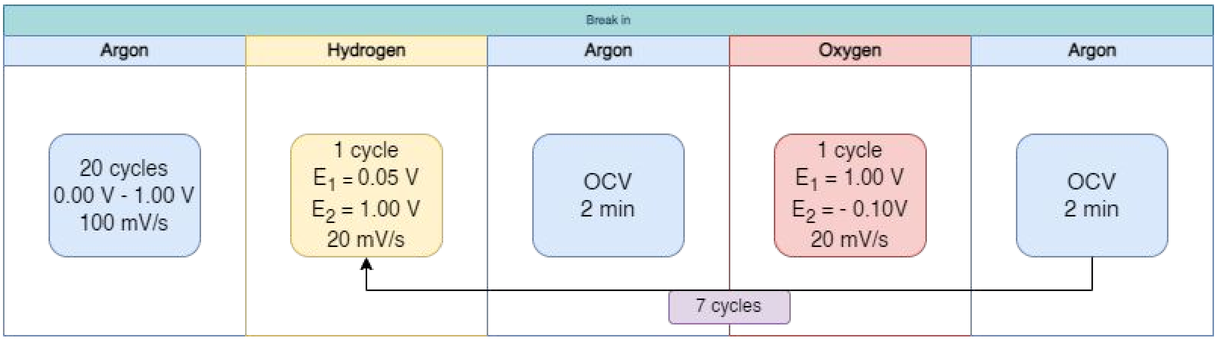

Scheme 1: Break-in protocol used as the activation protocol for the investigated electrocatalysts in MFE configuration.

S4. Particle size effect: the case of Pt/C analogues

In order to confirm/disprove the effect of particle size on ORR under MFE regime and to relate to the, according to our knowledge, the only available study on particle size effect under wide potential window (ref. 37 in the main text) two Pt/C analogues with different Pt paticle sizes were measured. Namely, apart from the reference sample i.e. an analogue with average particle size of 4.8 nm (refered here as Pt/C_4.8) another Pt/C analogue from the same producer (i.e. Tanaka Kikinzoku Kogyo, TEC10E50E-HT Pt/C) with an average particle size of 2.6 nm was measured for comparative analysis (refered to as Pt/C_2.6). Accordingly, the ORR_{spec} trend shows that a better performance is obtained for the case of larger particles from 0.8 V onwards cathodically (**Figure S7**). We note that this is in line with previosuly mentioned FET results (ref. 37 in the main text). Importantly, the obtained trend within the two Pt/C analogues additionally supports the beneficial effect of TiO_xN_y/C-platinum interaction.

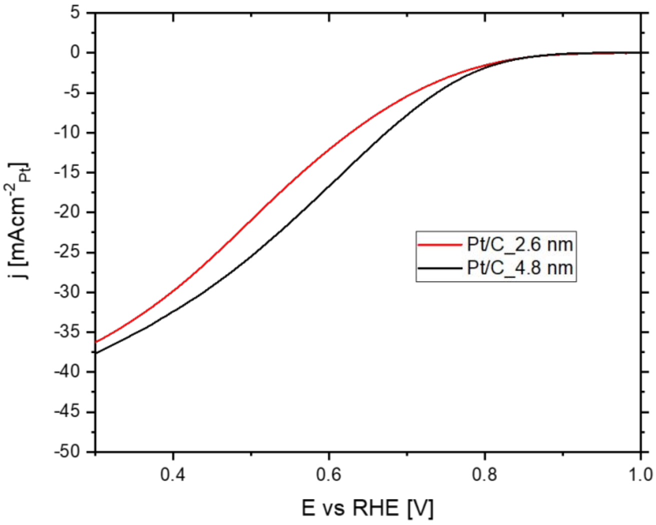

Figure S7: Comparison of ORR_{spec} via MFE for the two comercial Pt/C samples with different particle sizes (1 M $HClO_4$ 20 mV/s). Anodic scan is shown.

S5. CO stripping simulation (COSS) protocol

Typically CO_{stripp} is conducted via CO adsorption and stripping where the current used in subtraction from the CO_{stripp} polarization curve is taken as the second anodic scan. However, this might lead to erroneous determination of platinum surface area (HUPD and CO_{stripp} charges). Namely, certain oxide-based supports can get reduced under potentiostatic adsorption of CO. This means that the oxide-based support (the reduced form) electrochemically oxidizes in the subsequent scan. Hence it contributes to Faradayic response which is taken as the background current for subtraction. Instead, as shown in ref. [4] the proper background current should be measured in a separate measurement under the same protocol as CO_{stripp} (the so-called CO stripping simulation, COSS) with the following steps:

1. Potentiostatic treatment in the absence of CO.
2. Voltammetry in the absence of CO.

We have followed the COSS protocol and from comparative analysis it is clear that depending on the protocol used the surface areas obtained via HUPD charge substantially differentiate (**Table S1**). This should be ascribed to Faradayic contribution of the support (**Figure S8**).

Table S1. ECSA values depending on the protocol employed.

Catalyst	$ECSA_{CO}[m^2/g_{Pt}]$ non-COSS approach	$ECSA_{CO}[m^2/g_{Pt}]$ COSS approach	$ECSA_{HUPD}[m^2/g_{Pt}]$ non-COSS approach	$ECSA_{HUPD}[m^2/g_{Pt}]$ COSS approach
Pt-TiO_xN_y/C	68.71+-	63+-	51.58+-	63+-

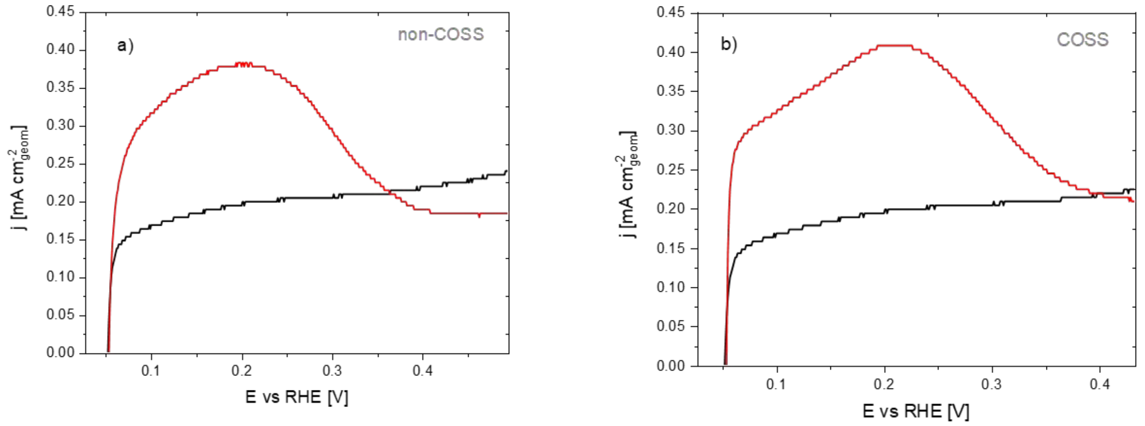

Figure S8: CO_{stripp} response for the Pt-TiO_xN_y/C sample using TF-RDE technique (20 mV/s). **a)** HUPD peak (red) can be taken as the second cycle and corrected with the first cycle (black). **b)** Alternatively, HUPD can be obtained in a separate experiment via the COSS protocol (red) and corrected with the first cycle from CO_{stripp} (black). See Experimental for detailed description of the COSS protocol.

S6. Loading independent region in MFE measurements

From **Figure S9** it is clear that for the MFE measurements for each sample, there exists a range of Pt loadings that measured gives the intrinsic catalytic activity of the sample. Note that for the Pt/C analogue, this loading-independent regime was already determined in our recent publication.[5] We emphasize that for each sample the ORR analysis was conducted in the loading-independent ORR_{spec} regime ensuring an intrinsic comparison of ORR performances (i.e., without additional mass transport effects).[5,6] For the Pt-TiO_xN_y/C sample this loading-independent region is measured to be between the 7.8 $\mu g_{Pt}cm^{-2}$ and 9.1 $\mu g_{Pt}cm^{-2}$. We believe that for the Pt loadings larger than the loading independent region, the part of the catalyst is not in contact with the electrolyte during ORR due to the larger film thickness, and at lower loading, bad homogeneity of the film lowers the performance of the ORR reaction.

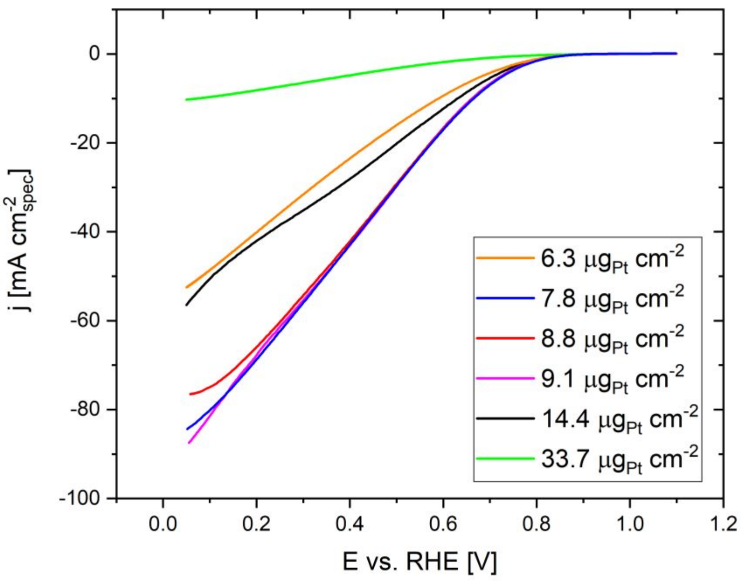

Figure S9: ORR polarization curves of Pt-TiO_xN_y/C sample showing different specific activities for a series of Pt loadings.

S7. O/OH adsorbate coverage state comparison

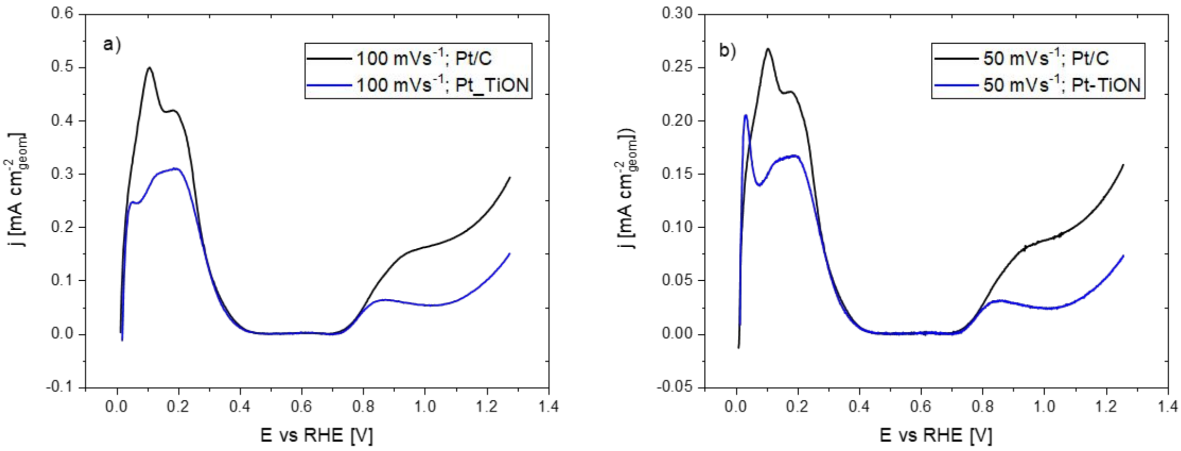

Figure S10: Blank cyclic voltammetric response for the two analogues investigated (anodic scan is shown only). Curves normalized in terms of geometric surface area and corrected for the background current from the double-layer capacitance. Measurements are recorded in 0.1 M $HClO_4$. **a)** Sweep rate of 100 mV/s. **b)** Sweep rate of 50 mV/s.

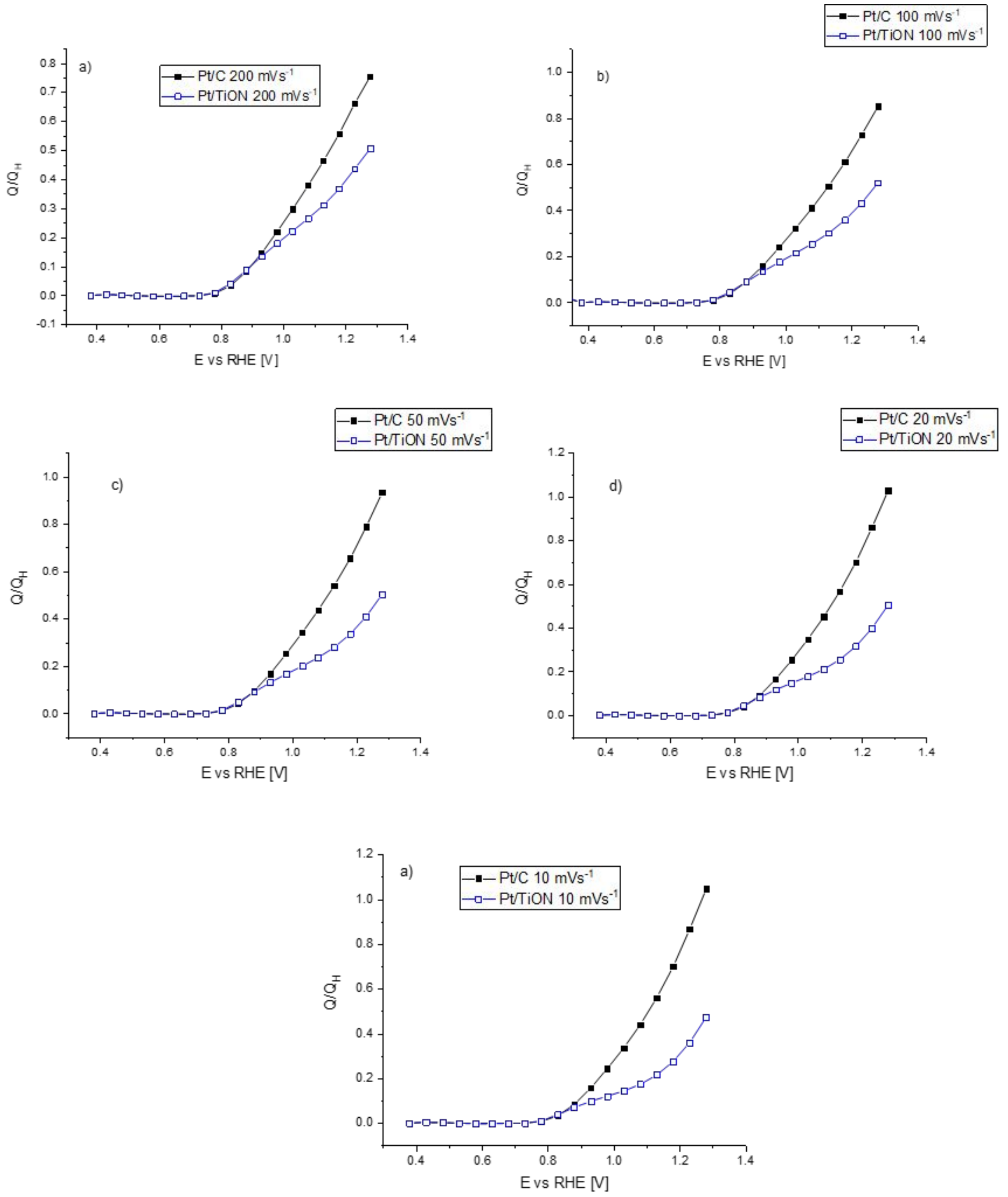

Figure S11: Apparent coverage of oxygen species on the Pt surface for both Pt/C and Pt-TiO_xN_y/C samples recorded at different potential sweep rates.

S8. Accelerated stress tests (AST) under MFE regime

In order to verify whether the titania-based encapsulation layer develops during prolonged electrochemical perturbation as observed in certain cases of titania-based supports[7–9] we performed accelerated stress tests (AST) for the Pt-TiO_xN_y/C analogue under MFE configuration. The protocol consisted of 10 000 cycles and performed in the potential range 0.6-0.95 V in Ar-purged atmosphere at a scan rate of 100 mV/s. Here the main goal was to pursue ORR performance trend and CO_{stripp} response before and after AST protocol. Namely, if an encapsulation layer was to develop over prolonged cycling

this should manifest in ORR polarization as well as in CO_{stripp} response. Namely, both reactions should be inhibited due to the layer's impermeable character for O_2 and CO.[7,10–12] According to our results this is evidently not the case, where both reactions stay virtually unaltered indicating on the absence of the encapsulation layer, at least not to a significant extent **(Figure S12)**.

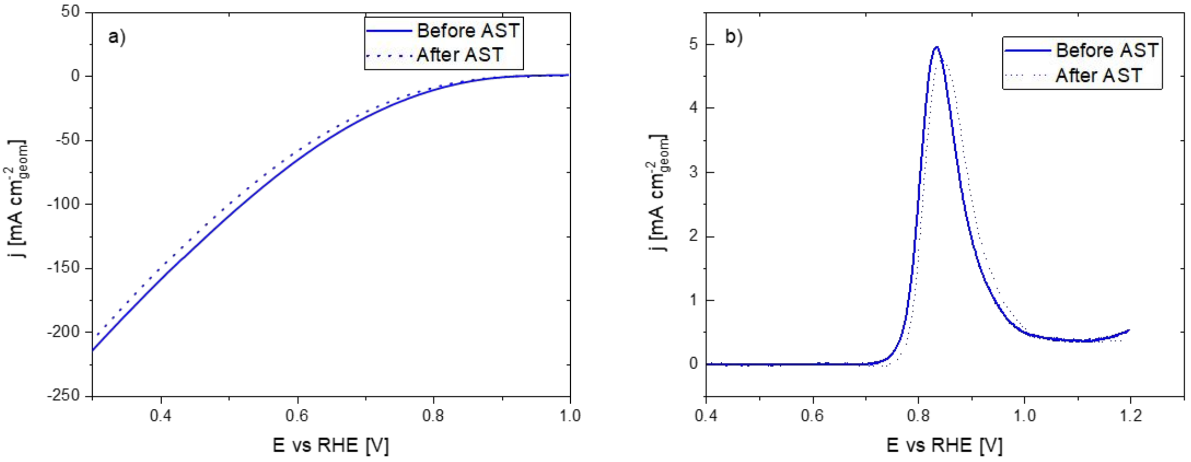

Figure S12: a) ORR polarization curves measured prior (solid line) and after (dash line) AST protocol. Measurements are recorded with the potential sweep rate of 20 mV/s. **b)** CO_{stripp} response measured prior (solid line) and after (dash line) AST protocol corrected for the background current from the double-layer capacitance. Measurements are recorded with the potential sweep rate of 50 mV/s.

S9. Hydrogen oxidation reaction (HOR)

Additional measures exploited HOR to confirm or dispute the existence of the above mentioned encapsulation layer. Namely, as shown in the past H^+ and H_2 transport are possible through the thin surface titania-based film whereas the transport of oxygenated compounds is impeded. Consequently such composites have a unique property to sustain the proceeding of HOR within atypical potential window i.e. at potentials where Pt is usually covered with oxygenated species.[7,11] Accordingly, we have used HOR as surface probe to inspect the response for the case of Pt-TiO_xN_y/C analogue. The obtained HOR polarization curve in general manifests similar behaviour as expected for Pt/C analogues i.e. HOR activity rapidly declines with the initiation of the Pt oxide region (**Figure S13**). Note that, the same HOR performance is obtained also after AST protocol. This HOR trends support the conclusion that Pt surface is most likely predominantly non-encapsulated which is also in line with ORR trends (see upper section). Nevertheless, the obtained HOR trend does demonstrate somewhat anomalous performance at high potentials where better performance is obtained for analogue in comparison to Pt/C. This confirms that a fraction of Pt surface is less covered with oxygenated adsorbates in Pt-TiO_xN_y/C which is also in close agreement with surface coverage analysis (see **Figure 6a** in the main text).

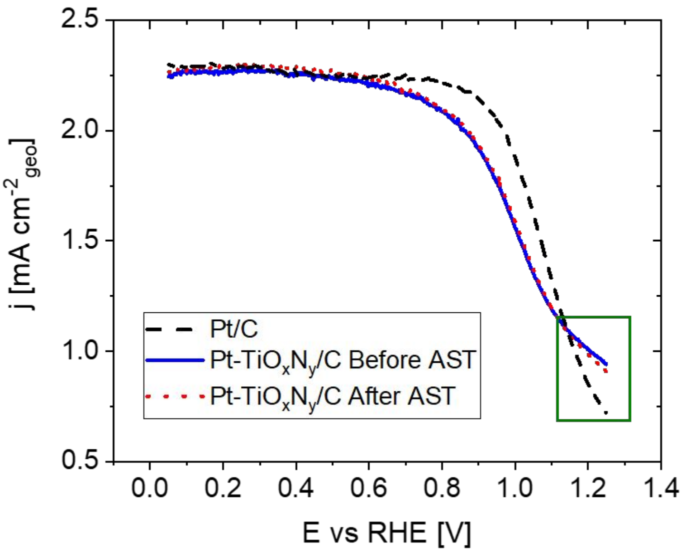

Figure S13: HOR polarization curves of Pt-TiO_xN_y/C and Pt/C recorded in H_2-saturated 0.1 M $HClO_4$ (10 mV/s, 1600 rpm, potential window 0.05 V vs. RHE – 1.25 V vs. RHE) collected for Pt-TiO_xN_y/C (blue) before and (red dash) after AST (protocol; 5000 CV scans, 300 mV/s, 0.6 V vs. RHE – 0.95 V vs. RHE). Pt/C polarization curve for comparison (black).

S10. Identical location transmission microscopy (IL-TEM)

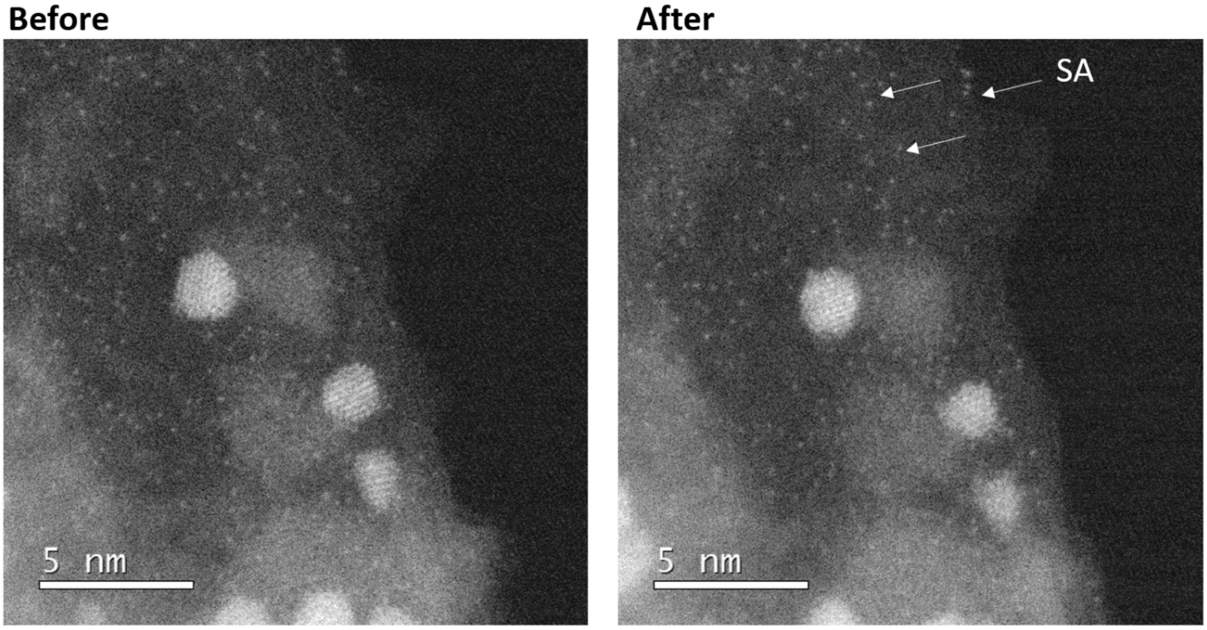

Figure S14: IL-STEM-HAADF images showing the presence of single atoms (SA) in Pt-TiO_xN_y/C, before and after the EP protocol.

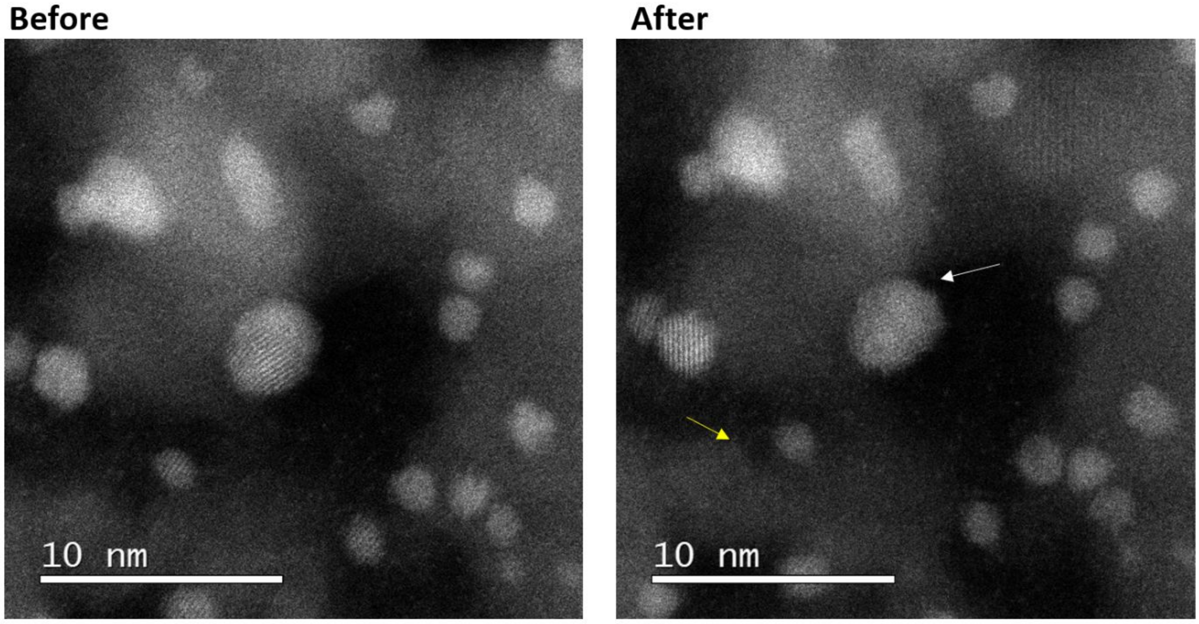

Figure S15: IL-STEM-HAADF images of before and after the EP protocol performed in Pt-TiO_xN_y/C showing the growth of material in some regions of the support (yellow arrow) and the corrosion of the supporting in other areas (white arrow).

References

(1) Smiljanić, M.; Panić, S.; Bele, M.; Ruiz-Zepeda, F.; Pavko, L.; Gašparič, L.; Kokalj, A.; Gaberšček, M.; Hodnik, N. Improving the HER Activity and Stability of Pt Nanoparticles by Titanium Oxynitride Support. *ACS Catal.* **2022**, *12* (20), 13021–13033. https://doi.org/10.1021/acscatal.2c03214.

(2) Marcano, D. C.; Kosynkin, D. V.; Berlin, J. M.; Sinitskii, A.; Sun, Z.; Slesarev, A.; Alemany, L. B.; Lu, W.; Tour, J. M. Improved Synthesis of Graphene Oxide. *ACS Nano* **2010**, *4* (8), 4806–4814. https://doi.org/10.1021/nn1006368.

(3) Lin, X.; Zalitis, C. M.; Sharman, J.; Kucernak, A. Electrocatalyst Performance at the Gas/Electrolyte Interface under High-Mass-Transport Conditions: Optimization of the "Floating Electrode" Method. *ACS Appl. Mater. Interfaces* **2020**, *12* (42), 47467–47481. https://doi.org/10.1021/acsami.0c12718.

(4) Binninger, T.; Fabbri, E.; Kötz, R.; Schmidt, T. J. Determination of the Electrochemically Active Surface Area of Metal-Oxide Supported Platinum Catalyst. *J. Electrochem. Soc.* **2014**, *161* (3), H121–H128. https://doi.org/10.1149/2.055403jes.

(5) Hrnjić, A.; Ruiz-Zepeda, F.; Gaberšček, M.; Bele, M.; Suhadolnik, L.; Hodnik, N.; Jovanovič, P. Modified Floating Electrode Apparatus for Advanced Characterization of Oxygen Reduction Reaction Electrocatalysts. *J. Electrochem. Soc.* **2020**, *167* (16), 166501. https://doi.org/10.1149/1945-7111/abc9de.

(6) Zalitis, C.; Kucernak, A.; Lin, X.; Sharman, J. Electrochemical Measurement of Intrinsic Oxygen Reduction Reaction Activity at High Current Densities as a Function of Particle Size for Pt 4– x Co x /C (x = 0, 1, 3) Catalysts. *ACS Catal.* **2020**, *10* (7), 4361–4376. https://doi.org/10.1021/acscatal.9b04750.

(7) Hornberger, E.; Bergmann, A.; Schmies, H.; Ku, S.; Wang, G.; Drnec, J.; Sandbeck, D. J. S.; Ramani, V.; Cherevko, S.; Mayrhofer, K. J. J.; Strasser, P. In Situ Stability Studies of Platinum Nanoparticles Supported on Ruthenium − Titanium Mixed Oxide (RTO) for Fuel Cell Cathodes. **2018**. https://doi.org/10.1021/acscatal.8b02498.

(8) Eckardt, M.; Gebauer, C.; Jusys, Z.; Wassner, M.; Hüsing, N.; Behm, R. J. Oxygen Reduction Reaction Activity and Long-Term Stability of Platinum Nanoparticles Supported on Titania

and Titania-Carbon Nanotube Composites. *J. Power Sources* **2018**, *400* (March), 580–591. https://doi.org/10.1016/j.jpowsour.2018.08.036.
(9) Banham, D.; Ye, S.; O'Toole, A.; Lemke, A.; Eisenbraun, E. Unexpected Hydrogen Oxidation Selectivity of Pt/NbTiO2 Catalysts. *Nano Energy* **2016**, *27*, 157–166. https://doi.org/10.1016/j.nanoen.2016.06.055.
(10) Holewinski, A.; Linic, S. Elementary Mechanisms in Electrocatalysis: Revisiting the ORR Tafel Slope. *J. Electrochem. Soc.* **2012**, *159* (11), H864–H870. https://doi.org/10.1149/2.022211jes.
(11) Stühmeier, B. M.; Selve, S.; Patel, M. U. M.; Geppert, T. N.; Gasteiger, H. A.; El-Sayed, H. A. Highly Selective Pt/TiOx Catalysts for the Hydrogen Oxidation Reaction. *ACS Appl. Energy Mater.* **2019**, *2* (8), 5534–5539. https://doi.org/10.1021/acsaem.9b00718.
(12) Hsieh, B. J.; Tsai, M. C.; Pan, C. J.; Su, W. N.; Rick, J.; Chou, H. L.; Lee, J. F.; Hwang, B. J. Tuning Metal Support Interactions Enhances the Activity and Durability of TiO2-Supported Pt Nanocatalysts. *Electrochim. Acta* **2017**, *224*, 452–459. https://doi.org/10.1016/j.electacta.2016.12.020.
